# A Conditional Structural Organization of the Light-Baryon Spectrum: Eighteen Channels and Nucleon Mass Ratios

**DOI:** 10.3390/e28091021

**Published:** 2026-09-13

**Authors:** Bin Li

**Affiliations:** Research Department, Silicon Minds Inc., Clarksville, MD 21029, USA; binli.siliconminds@gmail.com

**Keywords:** light-baryon spectrum, nucleon mass ratios, baryon centroids, quantum chromodynamics, information entropy, neutral-parent reconstruction, codimension-two defects, Fano plane, projective saturation, finite residue operators

## Abstract

Quantum chromodynamics (QCD) is the established dynamical account of light baryons. This paper asks whether their ground-state organization and dimensionless mass placement can also be represented by a finite reconstruction calculus. Given a three-grade rooted bilateral incidence complex, twenty-seven ordered routes reduce under the invariant side exchange to exactly eighteen classes. Complex linearization gives the standard representation identity ${\mathrm{Sym}}^{2}\left(W\right)⊗W≅{\mathrm{Sym}}^{3}\left(W\right)⊕S_{\left(2,1\right)}\left(W\right)$, with dimensions ten and eight. Identifying these sectors with the physical decuplet and octet is an explicit light-flavor correspondence postulate; the result is therefore a structural reorganization of known flavor combinatorics, not an independent derivation of the observed multiplets. A scale inherited from the published charged-lepton construction and finite operators then organize the masses. Data-informed closed-neutral and charged-boundary closures reproduce the neutron–electron and proton–electron ratios within 0.92 and 0.35 quoted experimental uncertainties. The leading seven-centroid comparison uses two common normalization integers and five effective discrete operator weights for seven values, so it is not an overdetermined prediction. Higher centroid and charge fibers were recognized with knowledge of the spectrum; their level-six agreements are consequently experimental-normalized reproducibility diagnostics, not statistical significances. A quadratic-local obstruction theorem excludes a simpler sector-blind charge selector, and the frozen carrier-lift complex gives a prospective Delta charge pattern. Published law–constant co-selection results motivate the common structural setting but do not prove the baryon-specific fibers. No continuously adjusted baryon-sector scale or measured fine-structure constant is inserted. The finite mathematics is exact within the declared complexes, while physical selection remains conditional; QCD and QED remain indispensable after read-out.

## 1. Introduction

The observed light-baryon ground states form a remarkably orderly spectrum. The spin-1/2 states organize into an octet and the spin-3/2 states into a decuplet, with approximate flavor relations and an almost regular decuplet spacing. Quantum chromodynamics (QCD) is the tested continuum theory of the strong interaction and provides the standard dynamical account of hadronic masses through quark and gluon fields, confinement, chiral dynamics, quark masses, and scale setting [1,2,3]. Nothing in this paper replaces that description.

The numerical motivation for asking an additional structural question is nevertheless substantial. Lattice-QCD calculations at the physical point typically reproduce the light octet and decuplet spectrum at the few-percent level, although selected stable-state calculations can attain considerably higher precision [4,5]. In the present construction, dedicated closed-neutral and charged-boundary fibers give the neutron–electron and proton–electron mass ratios, while the leading common baryon operator—before any higher residue is introduced—places all seven remaining centroids within 0.4% of their experimental values without fitting a continuous baryon-sector scale. This absence of a continuous fit is not equivalent to parameter-free prediction. At the operator level, the leading seven-centroid comparison contains two common normalization integers and five effective discrete weights, equal in number to the seven displayed values. The finite level-six operator reduces the largest relative non-nucleon centroid residual to approximately 0.021%, and the resolved non-Delta charge states show comparable numerical agreement, but the dedicated nucleon fibers and several refined exposure fibers were historically identified with knowledge of the spectrum. These results are therefore reproducibility and internal-consistency tests of a frozen finite construction, not a more accurate first-principles calculation than lattice QCD or a statistical discovery.

The two approaches therefore have complementary strengths and limitations. QCD supplies a complete dynamical theory, broad experimental coverage, and systematically controlled statistical, continuum, finite-volume, scale-setting, and quark-mass uncertainties. Its connection from the fundamental action to individual hadron masses, however, is necessarily nonperturbative and computationally demanding. The reconstruction approach seeks a different advantage: a finite, geometrically visualizable, and explicitly enumerable account of channel multiplicity and dimensionless mass placement. Its calculations are transparent once the incidence complexes and read-out premises are fixed, but their physical interpretation remains conditional, and a complete uncertainty theory has not yet been developed. Thus, QCD explains the dynamics of the read-out baryons, whereas reconstruction tests whether one finite architecture can organize the known channel family and its structural mass weights.

The question addressed here is therefore logically prior to a QCD or QED mass decomposition. Can one finite closure structure organize both the two nucleon ground states and the remaining light-baryon spectrum before one writes an effective Lagrangian with already identified fields, couplings, representations, and mass parameters? A Lagrangian is exceptionally powerful for propagation, scattering, decay, radiative response, and the decomposition of an already selected state. The premetric selection question proposed here is not formulated at that level because it asks which persistent objects and structural weights are admissible before the effective field variables have been chosen.

The proposal belongs to a reconstruction program in which particle labels, law forms, and dimensionless numerical normalizations are read-outs of a common premetric carrier–defect architecture [6,7,8,9,10]. The now-published law-selection analysis argues that the persistent defect and the equipped carrier are co-selected: the Indefinite Reconstruction Stability Principle (IRSP) constrains admissibility and descent, while the carrier supplies causal, conformal, metric, and holonomy structure [9]. The published fine-structure analysis applies the same codimension-two setting to a holonomy-capacity normalization [10]. This law–constant co-selection provides a prior reason to test finite capacity operators rather than isolated numerical identities; it does not prove the baryon-specific incidence fibers used below. Their selection and uniqueness remain independent burdens of the present model. Section 2 gives a compact translation of the few nonstandard terms, and Section 3 states the division of labor between reconstruction and effective Lagrangian physics.

The central baryon object is a confined bilateral pocket,

(1) $$B=\left[{\left(Z_{2}^{-}\to Z_{3}^{-}\right)}_{\mathrm{conf}}\right]\hat{⊕}\left[{\left(Z_{2}^{+}\to Z_{3}^{+}\right)}_{\mathrm{conf}}\right],$$

where $\hat{⊕}$ means that the two sides close only as a coupled object. A latent continuation seam completes the joint boundary but is not a free constituent in the static state.

The first main result concerns the number of primitive ground-state routes supported by this pocket. The two confined sides form an unordered conjugate pair, whereas the continuation seam is a distinguished protected role. Each of the three roles carries the three projective grades. When leading projective substitutions commute with transverse closure, the admissible route space is $G^{3}$. The complete invariant of a route is its continuation grade together with the unordered multiset of its two side grades. Quotient-first saturation therefore gives

(2) $$\Sigma_{B}^{\left(0\right)}≅G^{3}/S_{2},\;\left|\Sigma_{B}^{\left(0\right)}\right|=18.$$

After complex linearization, this space decomposes canonically into sectors of dimensions 10 and 8. The construction and proof are given in Section 8. Once a three-dimensional grade space and the exchange symmetry of the two side roles are posited, this is the familiar representation-theoretic 10+8 combinatorics. The observed decuplet and octet are used afterward to impose and test a light-flavor correspondence; the orbit calculation does not independently derive their physical identification.

The organizing hypothesis tested in this paper is that the complete intrinsic isolated mass is a saturated reconstruction invariant,

(3) $$M_{B,a}^{\mathrm{sat}}=R^{2}F_{B,a}^{\mathrm{sat}},$$

with no independent appearance of measured α. Here, *a* denotes a charge or boundary-orientation state. The primary statement is dimensionless:

(4) $$\frac{M_{B,a}^{\mathrm{sat}}}{m_{e}}=\frac{R^{2}}{m_{e}}F_{B,a}^{\mathrm{sat}},$$

and $m_{e}$ serves only as the external mass unit used to report values in MeV. The effective field-theory decomposition

(5) $$M_{B,a}^{\mathrm{phys}}\hat{=}M_{B,a}^{\mathrm{QCD}}+\delta M_{B,a}^{\mathrm{QED}}+\delta M_{B,a}^{m_{d}-m_{u}}+\cdots$$

remains legitimate and useful, but the hat over the equality emphasizes that it is a post-read-out representation of one completed physical state, not necessarily the ontological construction sequence of that state. In the reconstruction description, α and the baryon masses may be distinct read-outs of the same neutral-parent architecture rather than independently adjustable inputs.

Seven developments organize the construction. First, premetric equal weighting is stated as a maximum-entropy consequence of permutation invariance on a finite admissible route set. Second, the common three-grade embedding is defined through the canonical filtration and mod-2 cohomology of ${\mathrm{RP}}^{2}$. Third, an explicit rooted incidence complex and its complete invariant ledger yield an exact eighteen-class orbit theorem, whose physical light-flavor interpretation is stated separately. Fourth, under an explicit independence assumption at the leading mass-orientation level, the two confined sides inherit all three grades as six binary slots and hence give the conditional capacity $2^{6}=64$. Fifth, the neutron and proton are resolved as, respectively, the lowest closed-neutral realization and the lowest charged-boundary opening of the common baryon pocket. Their three correction indices follow conditionally from the explicitly stated binary-projective overlap read-out on the Fano incidence geometry of the three-grade space. Sixth, the remaining family coefficients and the first three centroid residues are assembled into finite operators on the same canonical 10+8 route space; their spectra replace unrelated numerical assignments by one operator calculation. Seventh, the charge problem is reduced to an isospin-path boundary problem. A no-go theorem excludes a sector-blind quadratic selector, while a finite carrier-lift complex gives the surviving octet/decuplet-conditioned source cochain and a new conditional Delta charge pattern.

**Remark** **1** (Principal contributions and claim status)**.** *The exact results are finite mathematical statements inside declared incidence complexes: the eighteen-channel quotient, the canonical 10+8 decomposition, the centroid-residue spectrum, the no-extra-motif classification, the isospin-path boundary theorem, and the carrier-source-cochain calculation. Their application to physical baryons is conditional on the stated reconstruction and read-out premises. In particular, the 10+8 identity reorganizes standard light-flavor combinatorics once the three-grade carrier and the side-exchange quotient are assumed; the map from that carrier to the physical flavor triplet is a correspondence postulate rather than an output of the orbit count. Historically, the small integer ledgers helped reveal the relevant exposure fibers; therefore, the refined numerical agreement is evidence for internal consistency rather than a blind statistical discovery. The leading centroid comparison is reported separately so that the scale and gross operator spectrum can be inspected before those higher residues are introduced; its discrete input count is stated explicitly in Section Input, Information, and Predictive-Status Audit. Dedicated neutron and proton formulas are treated as part of the baryon program. A conditional theorem derives their three correction indices from the unique binary projective plane once the explicit binary-projective overlap read-out is accepted; deriving that read-out itself from the complete premetric carrier calculus remains a theorem target. The Delta charge vector is an out-of-table consequence of the frozen combined source operator. These results support a conditional structural organization and retrospective reconstruction, not a parameter-free blind prediction.*

The paper is organized as follows. Section 2 gives a terminology guide, and Section 3 explains the division of labor between reconstruction and effective Lagrangian physics. Section 4 gives the finite maximum-entropy weighting rule, and Section 5 audits every assumption and conclusion. Section 6, Section 7 and Section 8 construct the primitive complex and prove the eighteen-class orbit theorem before the physical flavor correspondence is imposed. Section 9, Section 10, Section 11 and Section 12 develop the common mass normalization and refinement calculus. Section 13 and Section 14 construct the leading and centroid-residue operators. Section 15 derives the charge source and its Delta consequence. Section 16 develops the resolved proton and neutron ground-state closures. Section 17 reports the nucleon, centroid, and charge-resolved comparisons with experimental uncertainties and clearly labeled experimental-normalized residuals. Section 18 states the resulting conditional saturated-spectrum conjecture, Section 19 positions the proposal relative to QCD and other structural approaches, and Section 20 lists explicit failure criteria and remaining theorem targets.

## 2. Terminology and Reading Guide

Only a small vocabulary is needed for the finite calculations. The terms in Table 1 are operational labels, not replacements for standard field-theory quantities; their general development is given in Refs. [6,7,9]. In particular, “premetric” means logically prior to an effective metric read-out, not outside physics, and an arrow between structural stages denotes an admissible manifestation rather than a decay process.

## 3. Physical Background: Reconstruction, Persistent Identity, and Mass Read-Out

This section introduces the organizing spine of the construction. The framework begins with a neutral codimension-two archetype rather than with a separate premetric construction for each particle species. Different particles are interpreted as inequivalent completed embeddings and read-outs of this common structure in the carrier. The premetric construction selects the persistent identity and its structural weights, the read-out map represents that identity as an effective particle state, and the appropriate Lagrangian then governs its propagation and interactions after readout. Keeping these stages distinct prevents a structural selection rule from being mistaken for a substitute for QCD, QED, or the Standard Model.

### 3.1. Reconstruction and Effective Dynamics

Reconstruction is a logical and admissibility order, not a numerical time-stepping simulation of a spacetime that already exists. It asks whether a candidate local relation remains globally compatible under indefinite admissible continuation. A configuration that closes temporarily but cannot be continued consistently does not define a persistent physical identity.

Repeated compatible read-outs may nevertheless possess a smooth continuum limit. Ordinary time-dependent field equations are then the efficient coarse-grained description of small changes between successive read-outs. Let *P* denote a premetric reconstruction state, R an admissible reconstruction operation, and ρ the read-out map. If X=ρ(P) is the effective state, a regular reconstruction step may satisfy

(6) ρ∘R≃E∘ρ,

where *E* is an effective evolution law. This relation explains why continuum theories remain predictive: they compress stable relations among read-outs. It does not require a completed read-out to become ontologically independent of further reconstruction.

### 3.2. Why the Lagrangian Level Is Not the Selection Level

A Lagrangian description presupposes a spacetime arena, fields, representations, gauge groups, couplings, and a class of admissible states. It is therefore the correct language for propagation, scattering, decay, renormalization, and dynamical response. The present proposal asks a different question: which persistent state, closure type, and dimensionless structural weight are admissible before those effective variables have been selected?

At that earlier logical level, there is not yet a metric volume element, local time derivative, field amplitude, or action density from which a conventional spacetime Lagrangian could be formed. Requiring such a Lagrangian to perform the selection would therefore assume the very spacetime and field content that the reconstruction is intended to select. This is why the missing theorem targets in this paper are complete finite incidence and read-out derivations, not a hidden premetric QCD Lagrangian. Once the read-out has occurred, however, the Lagrangian description is both applicable and indispensable. The two descriptions are therefore complementary:

(7) $$\begin{array}{c} \mathrm{reconstruction}:\;\mathrm{admissible}\;\mathrm{identities}\;\mathrm{and}\;\mathrm{structural}\;\mathrm{weights}\\ ⇓\\ \mathrm{effective}\;\mathrm{fields}:\;\mathrm{propagation},\;\mathrm{interaction},\;\mathrm{and}\;\mathrm{decomposition}. \end{array}$$

An effective contribution proportional to α may consequently be part of a valid post-read-out mass decomposition without implying that measured α must appear as an independent input in the deeper mass-selection formula.

### 3.3. Law–Constant Co-Selection and Its Evidential Limit

The published native-law analysis places laws and numerical normalizations at two read-out levels of one completed support rather than treating either as an arbitrary input to the other [9]. Schematically,

(8) $$\left.\begin{array}{cc} \; & \mathrm{IRSP}\;\mathrm{and}\;\mathrm{defect}\;\mathrm{persistence}⟶\mathrm{admissible}\;\mathrm{identity}\;\mathrm{and}\;U(1)\;\mathrm{holonomy},\\ & \mathrm{equipped-carrier}\;\mathrm{persistence}⟶\mathrm{causal},\;\mathrm{conformal},\;\mathrm{and}\;\mathrm{metric}\;\mathrm{structure} \end{array}\right\}⟶\begin{array}{l} \mathrm{native}\;\mathrm{law}\;\mathrm{classes},\\ \mathrm{finite}\;\mathrm{read-out}\;\mathrm{capacities}. \end{array}$$

The same codimension-two carrier that supports the U(1) law structure is used in the published fine-structure analysis to define a completed holonomy capacity [10]. Laws and constants are therefore co-inherited from one carrier–defect architecture; the constant is not obtained by algebraically manipulating an already chosen field equation.

This broader framework reduces the arbitrariness of searching for isolated integer relations, but it is not a proof of the baryon formulas. The present paper must still define each finite complex, identify every data-informed choice, count its effective discrete inputs, and separate retrospective reconstruction from prospective consequences. Co-selection is relevant evidence only insofar as the same structures are reused without retuning and generate independent constraints such as Theorem 11.

### 3.4. Persistent Identity and Codimension-Two Support

A particle must remain distinguishable from its surrounding carrier under indefinite reconstruction. The author’s earlier topological work classified admissible reconstruction operations and identified codimension two as the critical local support for persistent loop-detectable obstruction. The reconstruction framework selects codimension-two support as the natural carrier of persistent loop-detectable obstruction under indefinite admissible continuation and finite repair capacity [7]. For smooth embedded supports, the corresponding local-to-global framework further identifies the conditions under which the normal meridian survives globally, admits an Abelian holonomy read-out, and remains preserved under admissible continuation maps [8]. The carrier-closure particle theory then applied this result to particle identity [6].

In a three-dimensional readable spatial slice, removing a codimension-two core gives

(9) $$R^{2}\setminus \left\{0\right\}≃S^{1}.$$

A small loop can therefore link the core and retain winding and holonomy under continuous deformation. Codimension one supplies separation rather than linking, while a codimension-three point has an $S^{2}$ link with trivial fundamental group. Codimension two is thus the first support on which a locally invisible core can remain globally readable through its complement.

### 3.5. Why U(1) Phase Appears First

The linking circle has $\pi_{1}\left(S^{1}\right)≅Z$. A one-dimensional unitary holonomy maps its winding generator into U(1). This establishes the availability of a loop phase before later particle and hadron labels are resolved. It does not by itself derive the full electromagnetic Lagrangian; it supplies the topological carrier on which a later electromagnetic read-out can act.

### 3.6. The Neutral Parent as the Common Particle Archetype

The codimension-two structure is organized by a neutral parent $P_{0}$. The parent is not an additional observable particle and is not already a proton, neutron, charged lepton, neutrino, or quark. It is the common premetric archetype from which these effective identities can emerge through inequivalent admissible embeddings into the carrier. Its first schematic particle-readable resolution is

(10) $$P_{0}⟶Z_{2}⊕\left(Z_{2}\to Z_{3}\right).$$

The first $Z_{2}$ denotes the common twofold Lorentz-readable level. The nested $Z_{3}$ denotes a hadron-supporting completion inside one such branch; it is not identified with the full $SU{\left(3\right)}_{C}$ Yang–Mills theory. Higher $Z_{n}$ interfaces are conditional refinements of an already selected state.

The essential physical picture is one archetype with multiple admissible routes, not a collection of unrelated hidden mechanisms. A completed route specifies how the same codimension-two identity is embedded, oriented, protected, and exposed to the carrier. Different routes can therefore produce different charge, mass, flavor, magnetic, and continuation read-outs. Repeated electrons or nucleons are spacetime realizations of the corresponding read-out channel; they are not separate copies of the premetric archetype.

In this sense, particle diversity arises through different manifestations of one structural map:

- A charged-lepton read-out exposes a transverse charged branch and its mass hierarchy;
- A neutral continuation read-out may retain protected content in the kernel of the scalar-mass map;
- A nucleon read-out distinguishes the lowest neutral closure from a charged boundary opening;
- A light-baryon read-out completes the two parent orientations as a confined bilateral pocket.

The codimension-two parent supplies the common identity spine, while the embedding and exposure pattern determine which physical sector is read out. This is why structural quantities obtained in one sector can be reused as inputs or consistency tests in another without identifying the resulting particles with one another.

### 3.7. Previous Cross-Sector Results and Motivation

The present baryon construction was not introduced in isolation. The same neutral-parent and codimension-two architecture now has published law-side and constants-side applications. Table 2 summarizes the results most relevant to the present extension; because the papers share structural premises, they form a dependency chain rather than four statistically independent confirmations.

These results span law, holonomy, magnetic, and inertial-mass read-outs and reuse the distinction between protected identity structure and exposed carrier residue. Their agreement does not prove the neutral-parent interpretation, and the numerical constructions used experimental regularities already known during discovery. They nevertheless show that the framework was not created solely for the present baryon spectrum. The law and fine-structure results motivate the common architecture; neither the magnetic-moment ratio nor measured α is used in the mass calculation. Only the published charged-lepton ratios determine the inherited cross-sector scale. The proton and neutron closures are then developed within the unified baryon analysis below.

### 3.8. Why the Baryon Is Bilateral and Confined

A baryon is modeled as a coupled closure of the two parent orientations. Neither side is independently complete; their boundaries close only when the positive side, negative side, and latent continuation seam are combined. This bilateral completion is the baryon-specific embedding of the common neutral-parent archetype.

Quarks and gluons remain the correct effective QCD variables after read-out. The premetric symbols used here do not denote smaller particles occupying an ordinary spatial interior. They label closure roles whose completed read-out is subsequently represented by the quark, gluon, flavor, and spin variables of QCD.

### 3.9. Why a Common Scale Is Tested

A genuine common-archetype theory should not require an unrelated mass scale for every sector. The baryon calculation therefore reuses the neutral-parent scale already fixed by the charged-lepton read-out and asks whether discrete baryon operators alone can place the spectrum. The construction uses

(11) $$R^{2}=\frac{m_{e}+m_{\mu }+m_{\tau }}{2}.$$

This choice is transparently the Koide combination, not an unrelated baryon-sector scale. The charged-lepton construction represents the Koide relation [11,12] as

(12) $$\frac{m_{e}+m_{\mu }+m_{\tau }}{{\left(\sqrt{m_{e}}+\sqrt{m_{\mu }}+\sqrt{m_{\tau }}\right)}^{2}}=\frac{2}{3}.$$

Consequently,

(13) $$R^{2}=\frac{1}{3}{\left(\sqrt{m_{e}}+\sqrt{m_{\mu }}+\sqrt{m_{\tau }}\right)}^{2}.$$

Thus, the scale used below is the Koide root-amplitude combination already fixed by the published charged-lepton sector. Equations (12) and (13) add no baryon datum; they disclose the identity connecting the inherited scale to that prior structural result. The symbol $R^{2}$, rather than *R*, is the scale used throughout this paper; it has the physical dimension of mass. Dividing by $m_{e}$ and using the published structural charged-lepton outputs

(14) $$r_{\mu }^{\mathrm{str}}=206.768282689,\;r_{\tau }^{\mathrm{str}}=3477.441636,$$

gives

(15) $$\frac{R^{2}}{m_{e}}=\frac{1+r_{\mu }^{\mathrm{str}}+r_{\tau }^{\mathrm{str}}}{2}=1842.6049593445.$$

The full charged-lepton tower and its neutral-seam correction are derived in Ref. [11]; measured muon or tau masses are not substituted into Equation (15). The electron mass supplies only the external unit used to report values in MeV. Within the reconstruction interpretation, $R^{2}$ is the neutral-parent first-resolution scale revealed especially cleanly by the charged-lepton sector. Its reuse here is therefore a cross-sector test rather than a fitted baryon-sector scale. It does not mean that lepton dynamics causes hadron masses.

### 3.10. From a Closure Weight to a Physical Rest Mass

The finite calculus produces a dimensionless signed factor $F_{C}$ for a completed closure class *C*. Comparison with a physical mass requires an explicit read-out premise rather than the declaration that a chamber count is itself a mass. The minimal premise used here is that the carrier maps $F_{C}$ to the coefficient of a normalized localized rest-energy profile,

(16) $$T_{C}^{00}\left(x\right)=R^{2}F_{C}\;\rho_{C}\left(x\right)+\mathrm{short-distance}\;\mathrm{derivative}/\mathrm{core}\;\mathrm{terms},\;\int d^{3}x\;\rho_{C}\left(x\right)=1.$$

When the derivative/core terms integrate to zero or are absorbed into the common normalization, the invariant rest energy is

(17) $$M_{C}=\int d^{3}x\;T_{C}^{00}\left(x\right)=R^{2}F_{C}.$$

After read-out, $M_{C}$ is the Lorentz scalar appearing in the ordinary worldline action

(18) $$S_{C}=-M_{C}c^{2}\int d\tau$$

and hence in the standard relativistic mass shell. This is a matching postulate between the premetric closure factor and the effective stress-energy description [13]; it is not a derivation of the QCD energy–momentum tensor or of confinement dynamics.

**Figure 1:**
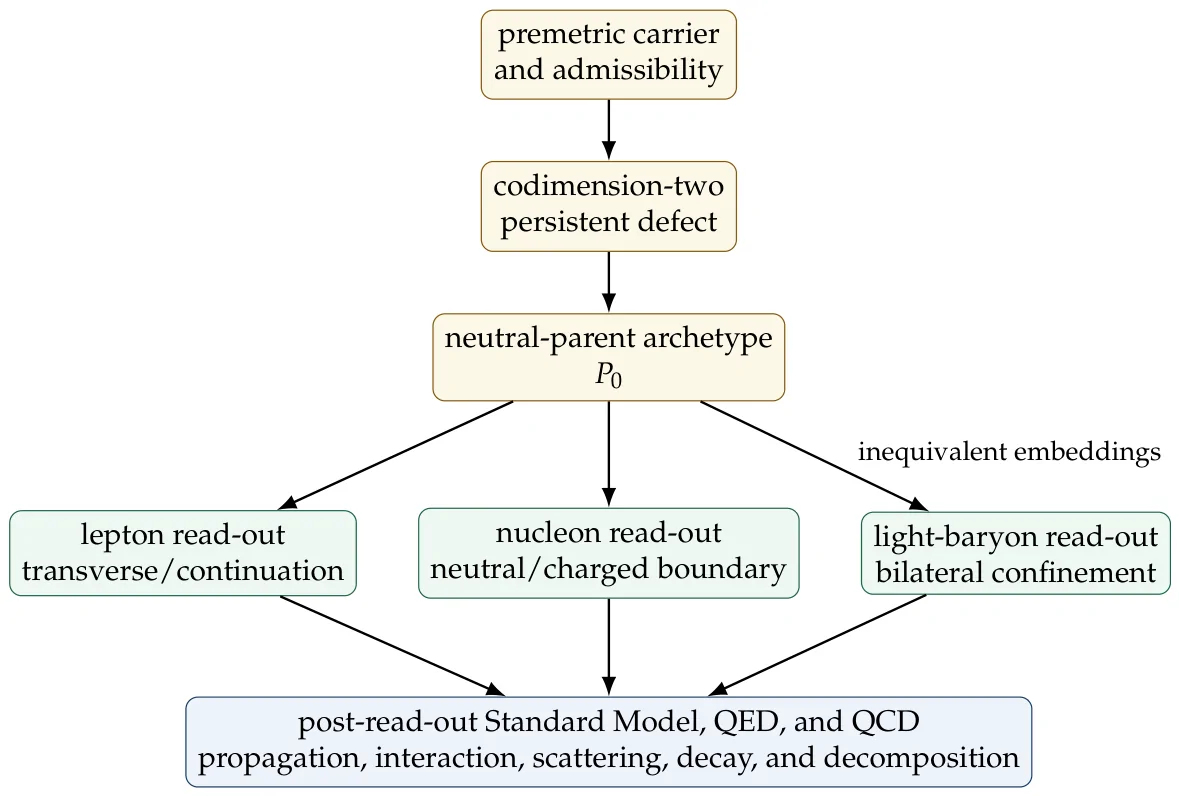
The neutral parent as the organizing spine of particle structure. Different particles are inequivalent completed embeddings and read-outs of the same codimension-two archetype, not separate premetric archetypes. Once a channel is read out, the appropriate Standard Model, QED, or QCD description governs its effective dynamics.

Figure 1 summarizes the proposed explanatory order. One neutral-parent archetype supports inequivalent particle read-outs, after which the Standard Model, QED, and QCD provide their effective dynamical descriptions.

## 4. Premetric Equal Weighting and Information Capacity

The finite counts used below are information-state counts, not thermodynamic microstate counts. Nevertheless, the equal-weight rule can be stated precisely with Shannon entropy rather than left as an informal symmetry intuition. Let Ω be a finite set of admissible premetric routes after all invariant distinctions have been retained and all representative redundancies have been quotiented. Suppose the automorphism group of the unresolved structure acts transitively on Ω. Before read-out, there is then no invariant datum that distinguishes one element from another.

The intuition is the same as labeling symmetry-related routes on a map before any route has acquired a physical name: assigning unequal weights would insert information that the premetric structure itself does not contain. Equal weighting is therefore a consequence of the unresolved symmetry once the admissible route set is fixed, not a claim that all post-read-out particles or energies are equal.

**Proposition** **1** (Permutation-invariant maximum-entropy measure)**.** *Let |Ω|=N<∞, and let a transitive automorphism group act on* Ω*. The unique invariant probability measure is*

(19) $$p_{\omega }=\frac{1}{N},\;\omega \in \Omega .$$

*It is also the unique maximizer of the Shannon entropy*

(20) $$H\left[p\right]=-\sum_{\omega \in \Omega }p_{\omega }\log p_{\omega },$$

*with*

(21) $$H_{\max}\left(\Omega \right)=\log N.$$

**Proof.**  Invariance under a transitive action forces all $p_{\omega }$ to be equal; normalization gives $p_{\omega }=1/N$. Strict concavity of Shannon entropy on the probability simplex makes this uniform distribution its unique maximum [14,15]. □

The inverse information capacity associated with an unresolved admissible route space is therefore

(22) $$q\left(\Omega \right):=\exp\left[-H_{\max}\left(\Omega \right)\right]=\frac{1}{N}.$$

This is the information-theoretic meaning of the inverse multiplicities that appear in the mass read-out. If independent refinement stages have route spaces $\Omega_{1},\ldots ,\Omega_{k}$, then

(23) $$H_{\max}\;\left(\prod_{j=1}^{k}\Omega_{j}\right)=\sum_{j=1}^{k}\log\left|\Omega_{j}\right|,\;q\;\left(\prod_{j=1}^{k}\Omega_{j}\right)=\prod_{j=1}^{k}\frac{1}{|\Omega_{j}|}.$$

Entropy additivity thus becomes multiplication of inverse-capacity denominators, matching the tower structure used later.

Uniformity can also be inherited by conditioning and products. If a finite route space carries the uniform measure, then conditioning on any nonempty subset assigns equal conditional weight to every retained route. Likewise, independently unresolved factors carry the product of their uniform measures. These observations justify later restrictions such as removing one protected identity chamber from an otherwise uniform permutation space. By contrast, when a later fiber combines structurally different roles and no transitive action is established, equal weighting is stated explicitly as part of the relevant read-out premise; it is not claimed as a consequence of symmetry alone.

The statement has a strict limitation. Maximum entropy fixes the weights after the admissible set and its invariant quotient have been defined; it does not determine which finite set nature admits. The eighteen-class orbit theorem below constructs its route set explicitly. The mass-exposure fibers and the nucleon binary-overlap read-out are separately declared and must ultimately be selected by a complete premetric carrier calculus. This separation prevents maximum-entropy language from concealing a data-informed choice of the underlying finite complex.

## 5. Claim Status and Scope

The paper combines standard topology, physical reconstruction postulates, conditional theorems, and baryon-specific ledgers. These levels are kept explicit.

1. **Standard mathematics.** A codimension-two defect has a transverse $S^{1}$ link; the unoriented line space in three dimensions is ${\mathrm{RP}}^{2}$; and
  (24) $$H^{*}\left({\mathrm{RP}}^{2};F_{2}\right)≅F_{2}\left[u\right]/\left(u^{3}\right),\;\deg u=1.$$
2. **Reconstruction principles.** Persistent identity requires return closure. IRSP first quotients reconstructions with no complete invariant distinction, and saturation then includes every inequivalent admissible channel required by closure. Deeper interfaces may refine a completed channel without creating a new primitive channel unless they introduce a new protected identity invariant.
3. **Information-theoretic consequence.** Once a finite admissible quotient is specified and its automorphism group acts transitively, permutation invariance and maximum Shannon entropy give equal weights 1/N. This determines the measure on the quotient, not the underlying admissible set.
4. **Exact finite orbit theorem.** Given the explicitly defined rooted bilateral incidence complex and leading grade-local completion, the primitive route spectrum is $G^{3}/S_{2}$, has exactly eighteen classes, and linearizes as canonical sectors of dimensions 10 and 8. Mapping those sectors to the physical light-flavor multiplets is a separate correspondence postulate.
5. **Other conditional mathematical consequences.** Given the stated physical identifications and independence assumptions, the construction yields the $2^{6}$ bilateral mass-orientation count. The first-refinement denominator 14 and the later *n*-tower additionally use the explicitly stated equal-weight saturation premises. Centroid preservation and absolute convergence then follow as shown below.
6. **Conditional leading-operator theorem.** Given the declared leading exposure fibers, one block-diagonal operator on the canonical 10+8 route space has eigenvalues
  (25) $$C_{B}=\left\{0,12,17,26;20,30,40,50\right\}$$
  on $N,\Lambda ,\Sigma ,\Xi ;\Delta ,\Sigma^{*},\Xi^{*},\Omega$.
7. **Conditional centroid-residue theorem.** An explicit finite signed exposure complex gives the first-refinement spectrum
  (0,0,4,−3;−1,4,6,−2),
  and two intrinsic projectors give the displayed Lambda and Sigma level-five and level-six terms. The complete displayed centroid ledger is therefore the spectrum of one finite operator rather than eight independent assignments.
8. **Conditional charge-source theorem.** An explicit carrier-lift complex and the canonical isospin edge primitives give the combined charge-orientation cochain through level six. The construction reproduces the resolved Sigma and Xi multiplets and gives an out-of-table Delta charge vector.
9. **Conditional nucleon correction-index theorem.** The closed-neutral and charged-boundary exposure counts give $N_{n}=470$ and $N_{p}=286$. Under the binary-projective overlap postulate, the three correction indices follow from normalized incidences in PG(2,2): 1/30 is a diagonal overlap, 3/7 is a closed-line density, and 23/42 is a line-stabilizer restoration index over the oriented flag fiber. Selection and uniqueness of that internal refinement remain premetric theorem targets.
10. **Spectrum conjecture.** The paper conjectures that the complete isolated baryon mass, including intrinsic charge-orientation refinement, is alpha-free at the reconstruction level. Effective QCD + QED decompositions remain valid after read-out.
11. **Phenomenological tests.** The leading spectrum is reported separately as a truncation test. The frozen displayed centroid operator is then compared with the seven non-nucleon centroids using quoted uncertainties and experimental-normalized residuals. The charge-source operator is compared with ten resolved non-Delta charge states, and its Delta vector is frozen as a conditional prediction. The dedicated proton and neutron formulas and their scale sensitivity are evaluated in the same calculation.

**Remark** **2** (What is and is not claimed)**.** *The paper constructs the eighteen-dimensional primitive channel space and proves its 10+8 representation split within the declared incidence complex. Identifying those sectors with the physical light-flavor decuplet and octet is a read-out correspondence. Likewise, the finite exposure and carrier-lift complexes produce exact operator spectra once their incidence relations are accepted, but a complete premetric carrier calculus has not yet proved that nature realizes precisely those fibers. This is therefore a conditional structural organization followed by a retrospective mass reconstruction. Its missing upstream results are incidence and read-out theorems, not a premetric version of the QCD Lagrangian. QCD remains the correct downstream theory of quark and gluon dynamics, chiral symmetry breaking, confinement, and effective mass decomposition.*

### Input, Information, and Predictive-Status Audit

Table 3 separates external inputs, prior structural results, conditional consequences, correspondence postulates, and empirical comparison data. This separation is essential: the absence of a continuous fitting parameter does not remove the need to state and test the discrete correspondence assumptions.

The external numerical information is limited and explicit. The measured electron mass is only the reporting unit; the two lepton ratios are prior published structural outputs that fix $R^{2}/m_{e}$; and measured baryon masses are comparison data that historically guided some fiber choices. Measured α is not used. Equal weighting follows only on a specified transitive quotient, and $z_{\exp}$ divides a residual by an experimental uncertainty without supplying a theoretical error model.

The operator-level count in Table 3 is deliberately conservative. Repeated cardinalities are counted where they enter as distinct signed or sector-conditioned coefficients, because the present theory has not yet proved that their placement and sign are forced. This does not assert that all entries are algebraically independent; it prevents shared small integers from concealing effective discrete model-selection freedom.

Consequently, “no continuously adjusted baryon-sector scale” is a true but limited statement. It does not imply zero effective model freedom. The refined mass agreements reported in Section 17 are reproducibility diagnostics for frozen finite complexes. The strongest independent content is instead the exact orbit structure, the finite operator identities, the obstruction Theorem 11, and prospective reuse without retuning—most immediately the Delta vector in Corollary 2.

## 6. Codimension-Two Identity and Projective Saturation

This section supplies the mathematical bridge between the physical picture and the later counts. The main idea is simple: a codimension-two object in a three-dimensional slice has a circular transverse link and an unoriented longitudinal axis. The circle governs return and phase; the axis space governs the leading projective classes.

Let *D* be the support of a primitive defect inside a three-dimensional carrier-readable spatial slice *M*. Assume

(26) $${\mathrm{codim}}_{M}D=2.$$

At a regular point x∈D, one has a local splitting

(27) $$T_{x}M≅T_{x}D⊕N_{x}D,\;\dim T_{x}D=1,\;\dim N_{x}D=2.$$

The two factors generate complementary structures.

**Theorem** **1** (Normal–projective decomposition)**.** *For a primitive unoriented codimension-two defect in a three-dimensional readable slice,*

*1.*  *The punctured normal fiber retracts onto a linking circle,*
  (28) $$N_{x}D\setminus \left\{0\right\}≃S^{1};$$
*2.*  *The unoriented tangent-axis space is*
  (29) $${\mathrm{Gr}}_{1}\left(R^{3}\right)≅{\mathrm{RP}}^{2}.$$

*Thus, the same parent supplies transverse loop holonomy and longitudinal projective embedding.*

**Proof.**  The normal space is two-dimensional, so removing the core gives $R^{2}\setminus \left\{0\right\}$, which deformation retracts onto $S^{1}$. The tangent direction is a line through the origin in $R^{3}$. Identifying opposite unit vectors n∼−n gives $S^{2}/\left(n∼-n\right)={\mathrm{RP}}^{2}$, equivalently the real Grassmannian of one-dimensional subspaces. □

The transverse $S^{1}$ supplies the primitive return generator, its two orientations γ and $\gamma^{-1}$, and finite cyclic resolutions. The longitudinal ${\mathrm{RP}}^{2}$ supplies the leading projective embedding grades.

**Lemma** **1** (Canonical projective grades)**.** *The canonical filtration*

(30) $${\mathrm{RP}}^{0}\subset {\mathrm{RP}}^{1}\subset {\mathrm{RP}}^{2}$$

*has one nonzero mod-2 relative class in each degree 0,1,2:*

(31) $$H_{k}\left({\mathrm{RP}}^{k},{\mathrm{RP}}^{k-1};F_{2}\right)≅F_{2},\;k=0,1,2.$$

*Here ${\mathrm{RP}}^{-1}:=⌀$ in the k=0 case. Equivalently, the cohomology ring has the three nonzero grades*

(32) $$1,\;u,\;u^{2},\;u^{3}=0.$$

**Theorem** **2** (Projective saturation theorem)**.** *Assume that leading $Z_{2}$-Lorentz embeddings are represented by the nonzero grades of the canonical projective filtration and that IRSP saturation realizes every independent complete grade without identifying different degrees. Then, the leading embedding set contains exactly three classes:*

(33) $$G=\left\{G_{0},G_{1},G_{2}\right\}≅\left\{1,u,u^{2}\right\}.$$

**Proof.**  By Equation (24), $1,u,u^{2}$ are nonzero and independent over $F_{2}$, while $u^{3}=0$. Saturation includes all three grades, and the non-identification assumption keeps them distinct. No fourth leading projective grade exists in ${\mathrm{RP}}^{2}$. □

**Remark** **3.** *The mathematical result is the existence of three leading projective grades. Their physical identification with the three observed fermion generations is a conditional carrier-resolution interpretation developed previously [6]. In the present paper, the grades are used more conservatively: they supply the three inherited bilateral orientation roles of each confined baryon side.*

**Figure 2:**
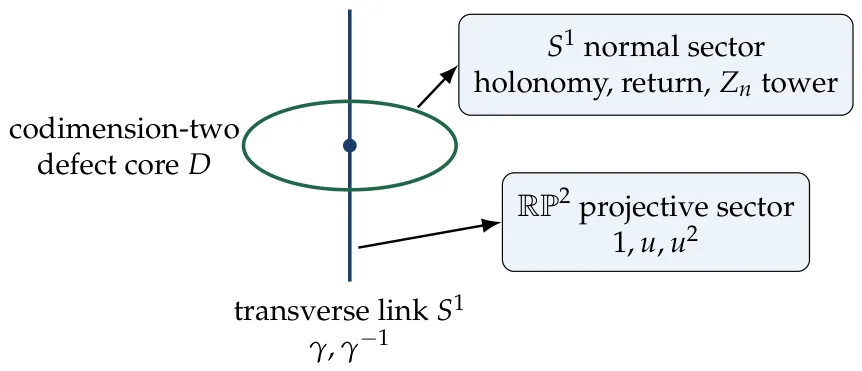
A codimension-two parent supplies two complementary structures. The transverse normal sector has linking space $S^{1}$, while the unoriented longitudinal axis has projective space ${\mathrm{RP}}^{2}$. The former controls return and higher interfaces; the latter supplies the three saturated leading grades.

Figure 2 displays the two structures used later in distinct roles: the transverse $S^{1}$ supplies return orientation, whereas the longitudinal ${\mathrm{RP}}^{2}$ supplies the three leading grades.

## 7. Confined Bilateral Baryon Pocket and Nonseparability

We now specialize the neutral-parent picture to baryons. In plain language, the proposed baryon is a closed pocket made from two oppositely oriented but individually incomplete sides. The purpose of the boundary-map notation below is to express that neither side is an observable baryon by itself.

The baryon pocket is the asymmetric confined realization of the neutral parent. Define

(34) $$B^{-}={\left(Z_{2}^{-}\to Z_{3}^{-}\right)}_{\mathrm{conf}},\;B^{+}={\left(Z_{2}^{+}\to Z_{3}^{+}\right)}_{\mathrm{conf}}.$$

The physical pocket is not $B^{-}⊕B^{+}$ as a direct sum of autonomous states. Introduce a closure boundary map

(35) $$\partial :C_{\mathrm{pocket}}⟶F_{\mathrm{seam}}.$$

**Principle** **1** (Bilateral closure)**.** *The two confined sides are individually incomplete,*

(36) $$\partial B^{-}\neq 0,\;\partial B^{+}\neq 0,$$

*but close jointly with a latent neutral-continuation seam $N_{\|}^{\mathrm{conf}}$:*

(37) $$\partial B^{-}+\partial B^{+}+\partial N_{\|}^{\mathrm{conf}}=0.$$

**Proposition** **2** (Bilateral nonseparability)**.** *Under Equations*(36) *and* (37)*, neither side defines an independently carrier-readable baryon, while*

(38) $$B=B^{-}\hat{⊕}B^{+}\hat{⊕}N_{\|}^{\mathrm{conf}}\in \ker\partial$$

*defines a completed confined pocket.*

The latent continuation seam is not counted as a free neutrino or an additional constituent mass. It is the continuation-direction component required for static whole-pocket closure. It may become externally readable only when the pocket opens through a weak process.

**Figure 3:**
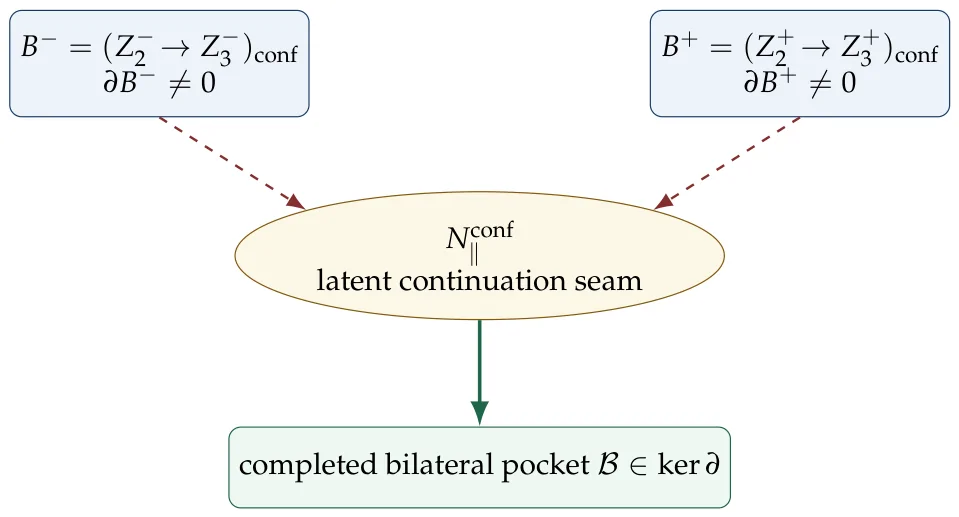
Bilateral nonseparability. Neither confined side closes independently. The latent continuation seam completes the total boundary without becoming a free constituent of the static mass formula.

Figure 3 illustrates this nonseparability: the completed pocket lies in ker∂ although neither side does so separately.

## 8. Primitive Baryon Incidence Complex and the Eighteen-Class Orbit Theorem

The preceding sections identify three structurally distinct roles in the completed pocket: two confined conjugate sides and one protected continuation seam. This section constructs their finite incidence complex and classifies its primitive route spectrum. The count is performed before the observed octet and decuplet are listed.

### 8.1. Map, Route, and Physical Realization

It is useful to distinguish three levels. The neutral parent supplies one structural “map”; an inequivalent completed path through that map is a primitive channel; and a particle occurring at a particular spacetime event is a post-read-out realization of a channel. Saturation completes every inequivalent admissible route. It does not count repeated realizations of the same route as new archetypes.

Formally, let $\mathrm{Adm}(P_{0})$ be the admissible routes of the parent and define

(39) $$x{∼}_{\mathrm{IRSP}}y\;⟺\;I\left(x\right)=I\left(y\right)\;\mathrm{for}\;\mathrm{every}\;\mathrm{complete}\;\mathrm{protected}\;\mathrm{or}\;\mathrm{readable}\;\mathrm{invariant}\;I.$$

The primitive channel spectrum is the quotient

(40) $$\Sigma \left(P_{0}\right)=\mathrm{Adm}\left(P_{0}\right)/{∼}_{\mathrm{IRSP}},$$

after which saturation requires every closure-supported class in $\Sigma (P_{0})$ to be represented. No minimization or stopping rule appears in this order of operations.

### 8.2. The Rooted Bilateral Incidence Object

The next definition extracts only the finite skeleton needed for the count. Its vertices are structural roles, not points in an already emergent space, and its edges record which roles meet the common closure root. Physically, the two side edges are interchangeable descriptions of one conjugate pair, whereas the continuation edge is distinguished by the job it performs in closing the whole pocket.

**Definition** **1** (Primitive rooted bilateral incidence complex)**.** *Let*

(41) $$V_{B}=\left\{v_{*},v_{1},v_{2},v_{\|}\right\},\;E_{B}=\left\{e_{1},e_{2},p_{\|}\right\},$$

*where*

(42) $$e_{1}=\left(v_{*},v_{1}\right),\;e_{2}=\left(v_{*},v_{2}\right),\;p_{\|}=\left(v_{*},v_{\|}\right).$$

*The edges $e_{1},e_{2}$ form an unordered conjugate side pair and $p_{\|}$ is the distinguished protected continuation incidence. The protected datum is*

(43) $$\eta_{B}=\left(p_{\|},\left\{e_{1},e_{2}\right\},\iota_{B}\right),$$

*with involution*

(44) $$\iota_{B}\left(e_{1}\right)=e_{2},\;\iota_{B}\left(e_{2}\right)=e_{1},\;\iota_{B}\left(p_{\|}\right)=p_{\|}.$$

*The resulting rooted incidence object is denoted $X_{B}=\left(V_{B},E_{B},\eta_{B}\right)$.*

The signs conventionally written on the two sides are relational orientations of the conjugate pair. Before carrier-orientation read-out there is no external datum that declares either side to be the first side. Accordingly, an isomorphism may exchange $e_{1}$ and $e_{2}$ while preserving their opposition relation and the protected root.

**Lemma** **2** (Automorphism group of the rooted pocket)**.** *The full incidence automorphism group preserving $\eta_{B}$ is*

(45) $$\mathrm{Aut}(X_{B},\eta_{B})=\{\mathrm{id},\iota_{B}\}≅S_{2}.$$

**Proof.**  Every allowed automorphism fixes $p_{\|}$ because it is the unique protected root. It must preserve the unordered two-element set $\left\{e_{1},e_{2}\right\}$. Hence, it either fixes both side incidences or exchanges them. Both permutations preserve the incidence relation and $\eta_{B}$, so the group is exactly $S_{2}$. □

**Figure 4:**
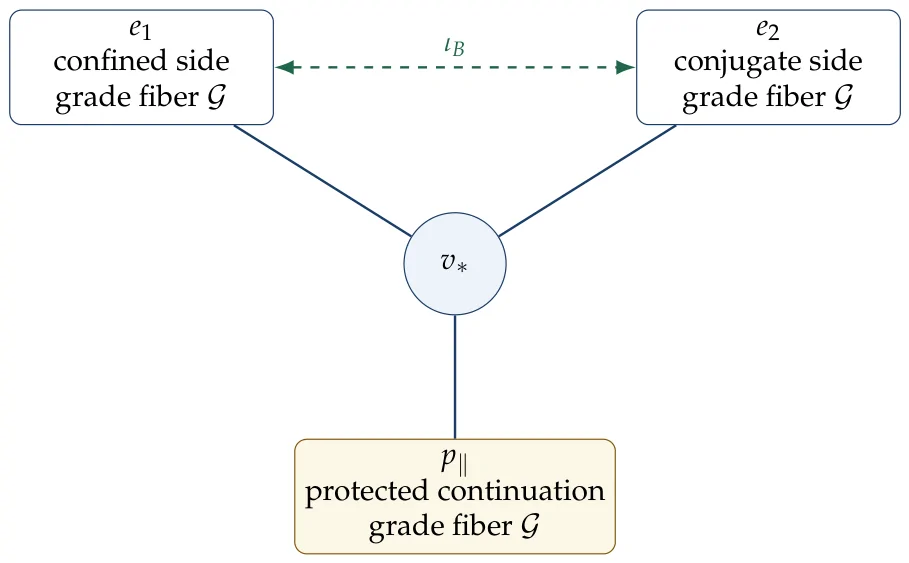
The primitive rooted bilateral incidence complex. The two confined-side incidences form one unordered conjugate pair and may be exchanged by $\iota_{B}$; the continuation incidence is protected and fixed. Each role carries the same three-grade projective fiber.

Figure 4 displays the rooted incidence complex and the only nontrivial representative symmetry used in the quotient.

### 8.3. Projective Decoration and Grade-Local Completion

The bare incidence skeleton says which roles are present but not which projective grade each role carries. Decorating the three edges therefore amounts to placing one of three admissible labels on each route segment. The grade-local assumption below says that changing one longitudinal label does not tear the transverse closure of the completed pocket; this is the precise premise that makes an exhaustive finite search possible.

Let

(46) $$G=\left\{g_{0},g_{1},g_{2}\right\}≅\left\{1,u,u^{2}\right\}$$

be the three projective grades established in Equation (33). A completed grade route is a coloring

(47) $$c:E_{B}⟶G,\;c=\left(g_{1},g_{2},g_{\|}\right).$$

The continuation seam is protected from direct scalar-mass exposure, but protection does not remove it from the complete identity ledger. IRSP continuation faithfulness therefore retains its projective grade distinction.

**Structural Postulate** **1** (Leading grade-local completion)**.** *At the primitive ground-state layer, the transverse boundary obligation and the longitudinal projective grade factor through distinct components of the normal–projective decomposition. Thus,*

(48) $$\partial_{B}\left(e,g\right)=\partial_{B}^{⊥}\left(e\right),\;e\in E_{B},\;g\in G,$$

*For an independent grade substitution $T_{e:g\to h}$, grade-local completion imposes the commutation relation*

(49) $${\mathrm{Comp}}_{B}\;T_{e:g\to h}=T_{e:g\to h}\;{\mathrm{Comp}}_{B}.$$

*No additional protected cross-grade constraint is present in the primitive datum $\eta_{B}$.*

This postulate is the finite leading-order form of the separation already used in Figure 2: the transverse $S^{1}$ controls return closure, whereas the longitudinal ${\mathrm{RP}}^{2}$ supplies projective grade. It is stated explicitly so that a hidden grade-coupling relation cannot be concealed in the count. Such a relation would invalidate the theorem below rather than being absorbed by a change of multiplicity.

**Lemma** **3** (Exhaustive raw route space)**.** *Under Structural Postulate 1, the completed primitive route space is*

(50) $${\mathrm{Adm}}_{B}^{\left(0\right)}=\mathrm{Map}\left(E_{B},G\right)≅G^{3},\;\left|{\mathrm{Adm}}_{B}^{\left(0\right)}\right|=27.$$

**Proof.**  There are three incidence roles and three allowed grades on each role. By Equations (48) and (49), changing the grade on one role preserves the completed boundary and does not constrain either of the other two grades. Hence, every map $E_{B}\to G$ is admissible, giving $3^{3}=27$ completed representatives. □

### 8.4. Complete Invariant Ledger and Quotient

The 27 colorings in Equation (50) are ordered representatives, not yet physical channel types. Since the two confined sides have no invariant first/second ordering, exchanging them must not create a new route. The ledger below retains exactly what survives that exchange: the protected continuation grade and the multiplicities of the three side grades.

For a route *c*, define the side-grade multiplicities

(51) $$m_{k}\left(c\right)=\#\left\{e\in \left\{e_{1},e_{2}\right\}:c\left(e\right)=g_{k}\right\},\;m_{0}+m_{1}+m_{2}=2,$$

and the invariant ledger

(52) $$I_{B}\left(c\right)=\left(c\left(p_{\|}\right);m_{0}\left(c\right),m_{1}\left(c\right),m_{2}\left(c\right)\right).$$

It records the continuation grade and the unordered multiset of the two side grades.

**Lemma** **4** (Completeness of the channel invariant)**.** *For $c,c^{\prime }\in {\mathrm{Adm}}_{B}^{\left(0\right)}$,*

(53) $$I_{B}\left(c\right)=I_{B}\left(c^{\prime }\right)\;⟺\;c^{\prime }=c\;\mathrm{or}\;c^{\prime }=\iota_{B}c.$$

*Consequently, the invariant ledger Equation (52) classifies the rooted colored complexes up to all allowed representative relabelings.*

**Proof.**  Equality of the first entry fixes the protected continuation grade. Equality of $\left(m_{0},m_{1},m_{2}\right)$ fixes the unordered two-element multiset of side grades. Two ordered presentations of the same two-element multiset are either identical or related by exchange of the side roles. Conversely, both the continuation grade and the side multiplicities are invariant under $\iota_{B}$. □

**Theorem** **3** (Premetric eighteen-class orbit theorem)**.** *For the primitive rooted bilateral complex $X_{B}$ with projective grade set G and grade-local completion, quotient-first saturation gives*

(54) $$\Sigma_{B}^{\left(0\right)}={\mathrm{Adm}}_{B}^{\left(0\right)}/{∼}_{\mathrm{IRSP}}≅G^{3}/\langle \iota_{B}\rangle ,$$

*and*

(55) $$\begin{array}{c} \left|\Sigma_{B}^{\left(0\right)}\right|=18. \end{array}$$

**Proof.**  By Lemma 4, the IRSP equivalence classes are exactly the orbits of the side-exchange action. The continuation role has three possible grades. The two side roles contribute an unordered multiset of size two drawn from three grades, of which there are

(56) 3+2−12=6.

Therefore, $|\Sigma_{B}^{\left(0\right)}|=3\times 6=18$.

Equivalently, Burnside’s lemma gives an independent check. The identity fixes all 27 routes. The exchange $\iota_{B}$ fixes precisely the routes with equal side grades; there are three choices for their common grade and three choices for the continuation grade, hence nine fixed routes. Thus,

(57) $$\left|G^{3}/\langle \iota_{B}\rangle \right|=\frac{27+9}{2}=18.$$

Saturation includes every one of these inequivalent classes; repeated representatives or repeated spacetime realizations do not add further primitive channels. □

For readers unfamiliar with orbit counting, the result can be pictured as three cards. One card records the continuation grade and remains distinguished. The other two record the confined-side grades, but exchanging those two cards does not change the pocket. There are three choices for the distinguished card and six unordered two-card selections with repetition, giving 3×6=18. Burnside’s lemma is the formal check that no representative is lost or counted twice.

### 8.5. Canonical Ten-Plus-Eight Decomposition

The integer eighteen is not only an orbit count. Linearization asks how the same declared route space transforms when complex superpositions are allowed. Symmetrizing the two interchangeable side factors produces ${\mathrm{Sym}}^{2}\left(W\right)⊗W$; the continuation factor remains distinguished. The ensuing 10+8 split is therefore an exact representation consequence of the chosen three-grade incidence symmetry. It is not, by itself, an empirical derivation of the physical octet and decuplet. Let

(58) $$W=C\left[G\right],\;\dim_{C}W=3,$$

be the free complex vector space on the three projective grades. Linearizing the side-exchange quotient gives

(59) $$V_{B}={\left(W^{⊗3}\right)}^{S_{2}}≅{\mathrm{Sym}}^{2}\left(W\right)⊗W.$$

**Corollary** **1** (Canonical decomposition of the declared route space)**.** *The primitive channel space decomposes as*

(60) $$V_{B}≅{\mathrm{Sym}}^{3}\left(W\right)⊕S_{\left(2,1\right)}\left(W\right),$$

*with*

(61) $${\dim\mathrm{Sym}}^{3}\left(W\right)=10,\;\dim S_{\left(2,1\right)}\left(W\right)=8.$$

*Hence, 18=10+8 canonically.*

**Proof.**  The first two tensor factors are symmetrized by the side exchange, giving Equation (59). The Pieri rule yields [16]

(62) $${\mathrm{Sym}}^{2}\left(W\right)⊗W≅{\mathrm{Sym}}^{3}\left(W\right)⊕S_{\left(2,1\right)}\left(W\right).$$

Since dimW=3,

(63) dimSym3(W)=3+3−13=10,

and the hook-length formula gives $\dim S_{\left(2,1\right)}\left(W\right)=8$. □

The two summands have a direct symmetry interpretation. The ten-dimensional part is unchanged by every permutation of the three roles; the eight-dimensional part retains the mixed pattern in which the two confined sides are symmetric but the continuation role remains distinguished. The labels “ten” and “eight” are therefore dimensions of symmetry sectors before they are named as physical flavor multiplets. Equivalently, the complete primitive kernel available to any downstream selector is

(64) $$K_{B}:=C\left[\Sigma_{B}^{\left(0\right)}\right]≅V_{B},\;\dim_{C}K_{B}=18.$$

This kernel statement is exact inside the declared complex. A later use of its dimension must separately justify why the new selector acts on $K_{B}$, is insensitive to representative labels, and weights each primitive class once.

### 8.6. Light-Flavor Read-Out and Observed Channel Content

The mathematics up to this point yields two canonical sectors of dimensions 10 and 8, but it does not by itself name them as physical flavor multiplets. The same 10+8 representation pattern is standard in flavor SU(3). The proposed contribution is a finite upstream realization of that familiar combinatorics; its physical use requires the following read-out correspondence, under which the three-dimensional projective grade carrier is represented as the effective light-flavor triplet after spacetime and QCD degrees of freedom have emerged.

**Structural Postulate** **2** (Sector-specific light-flavor read-out)**.** *At the post-reconstruction baryon read-out, the complex grade space is represented by the effective light-flavor triplet,*

(65) $$\rho_{F}:W⟶3_{F}.$$

*This is a sector-specific representation map. It does not identify light-quark flavor with fermion generation; it states that the same three-dimensional structural carrier is represented as the light-flavor triplet in this baryon sector.*

Under Structural Postulate 2, the two summands in Equation (60) read out as

(66) $${\mathrm{Sym}}^{3}\left(3_{F}\right)=10,\;S_{\left(2,1\right)}\left(3_{F}\right)=8.$$

The familiar ground-state channel list provides an independent physical check. The octet contains

(67) $$\left(p,n\right),\;\Lambda ,\;\left(\Sigma^{+},\Sigma^{0},\Sigma^{-}\right),\;\left(\Xi^{0},\Xi^{-}\right),$$

so $N_{8}=2+1+3+2=8$. The decuplet contains

(68) $$\left(\Delta^{++},\Delta^{+},\Delta^{0},\Delta^{-}\right),\;\left(\Sigma^{*+},\Sigma^{*0},\Sigma^{*-}\right),\;\left(\Xi^{*0},\Xi^{*-}\right),\;\Omega^{-},$$

so $N_{10}=4+3+2+1=10$. Thus, the observed matter-side ground sector has

(69) $$N_{\mathrm{light}\;\mathrm{baryon}}=N_{8}+N_{10}=18,$$

in the same 10+8 representation pattern as the declared route complex. This agreement checks the correspondence postulate; it is not an independent prediction of the already known multiplicities.

The theorem counts primitive channel types, not every post-read-out degree of freedom. Schematically,

(70) $$H_{\mathrm{post}}=\underset{\left[c\right]\in \Sigma_{B}^{\left(0\right)}}{⨁}V_{\left[c\right]}^{\mathrm{spin}}⊗H_{\left[c\right]}^{\mathrm{exc}}.$$

Magnetic spin projections and radial or orbital excitations lie in fibers over an already selected primitive channel. Confined color is not independently readable; antibaryons are charge-conjugate realizations rather than a second copy of the premetric map; and charm- or bottom-containing baryons belong to later effective flavor sectors. A future use of the number eighteen in a different observable must still prove that the corresponding selector factors through $\Sigma_{B}^{\left(0\right)}$ and weights each primitive class once.

**Remark** **4** (Proof boundary)**.** *Theorem 3 is an exact finite theorem within the declared primitive incidence complex and Structural Postulate 1. Its non-combinatorial burden is sharply localized: a complete premetric carrier construction must derive the commutation relation Equation* (49) *and exclude an additional protected cross-grade constraint. This is an upstream incidence problem, not a request for a spacetime Lagrangian. The flavor identification Structural Postulate 2 is a physical read-out correspondence, while the dimensions 18=10+8 are obtained before that correspondence is imposed.*

## 9. Bilateral Projective Orientation Space and the Denominator 64

The denominator 64 is the leading normalization of the baryon count. It is not introduced because it happens to fit the spectrum. It arises conditionally from two confined sides, three projective grades on each side, and a binary orientation choice for each grade.

Each confined side inherits the three saturated projective grades Equation (33). Let

(71) S={−,+}

be the two-side set and define the six bilateral role slots

(72) $$O_{B}=S\times G.$$

Then,

(73) $$|O_{B}|=2\cdot 3=6.$$

The transverse codimension-two linking generator has two orientations, represented by $Z_{2}=\left\{+,-\right\}$ at the finite read-out level.

**Definition** **2** (Leading bilateral orientation space)**.** *The leading orientation assignment space is*

(74) $$B_{0}=\mathrm{Map}\left(O_{B},Z_{2}\right)≅{\left(Z_{2}\right)}^{6}.$$

**Theorem** **4** (Bilateral projective-orientation theorem)**.** *Assume that the two confined sides inherit the three saturated projective grades independently at the leading orientation level and that each grade admits the two orientations of the primitive linking generator. Then,*

(75) $$|B_{0}|=2^{2\cdot 3}=2^{6}=64.$$

*If no leading orientation assignment is preferred premetrically, the normalized chamber weight is 1/64.*

**Proof.**  There are six independent binary slots in Equation (72). The number of maps from a six-element set to a two-element set is $2^{6}$. Uniformity follows from invariance under the translation action of ${\left(Z_{2}\right)}^{6}$ on itself. □

The independence assumed here is independence of orientation labels *within the completed joint pocket*; it is not independence of the two confined sides as observable particles. Bilateral closure first fixes the admissible whole, after which the six inherited grade slots label orientations of that whole. A complete premetric closure complex must verify that no additional joint-boundary relation further quotients this assignment space.

In elementary terms, the count is a six-switch ledger: two confined sides times three grades give six internal yes/no orientation slots. The completed baryon is still one object; the 64 entries enumerate the possible settings of those six internal switches, not 64 constituents or 64 particle species.

This theorem gives the leading baryon chamber quantum

(76) $$Q_{B}=\frac{R^{2}}{64}.$$

It also clarifies the meaning of the denominator: 64 is not a fitted scale and not the dimension of a postulated internal Euclidean space. It is the finite capacity of the saturated bilateral orientation assignments inherited from the codimension-two parent.

**Figure 5:**
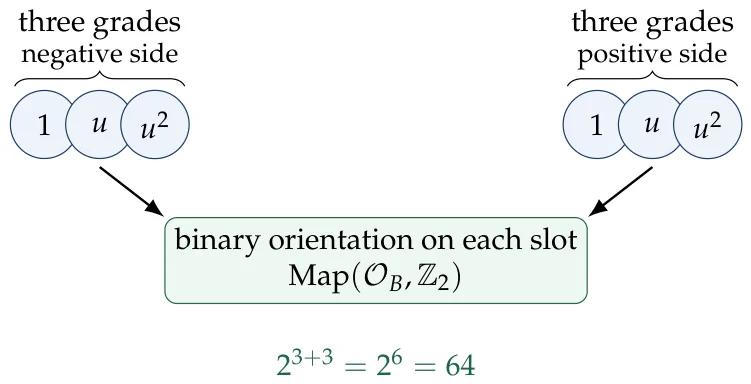
Derivation of the leading bilateral denominator. Each of the two confined sides inherits the three projective grades, and each slot admits two loop orientations.

Figure 5 summarizes the conditional counting that produces the six slots and the capacity $2^{6}$.

## 10. Rooted Five-Role Exposure and the Protected Ground-State Offset

The ground subtraction is the smallest term in the leading formula, but it requires careful interpretation. The five-role chamber space counts ordered read-out alignments around a rooted and oriented return loop. One trivial alignment preserves the existing identity and is therefore non-facing; the remaining alignments form the exposure space used in the normalization. The term *ground-state offset* refers to this internal subtraction and should not be confused with an external mass-reference anchor, which is only a choice of units.

The leading orientation space fixes the gross bilateral quantum. A separate protected subtraction fixes the confined baryon ground-state baseline. The relevant positive-side exposure is modeled by five distinct roles:

(77) $$A_{5}=\left\{1,u,u^{2},\varepsilon ,\eta \right\}.$$

Here, $1,u,u^{2}$ are the inherited projective grades, ε is the first carrier-facing exposure role, and η is the first confined positive-side completion role.

The linking loop is rooted at the carrier-facing seam and oriented by the selected traversal. Therefore, its positions are sequentially distinguishable; one does not quotient by cyclic rotation or orientation reversal. The ordered chamber set is

(78) $$C_{5}=\mathrm{Bij}\left(\left[5\right],A_{5}\right),\;\left|C_{5}\right|=5!.$$

There is one order-preserving identity chamber. Define

(79) $$C_{5}^{\times }=C_{5}\setminus \left\{\mathrm{id}\right\},\;\left|C_{5}^{\times }\right|=5!-1=119.$$

The completed confined $Z_{3}$ closure supplies three equivalent orientation sectors, so

(80) $$X_{\mathrm{anchor}}=Z_{3}\times C_{5}^{\times },\;\left|X_{\mathrm{anchor}}\right|=3\left(5!-1\right)=357.$$

The position-permutation action of $S_{5}$ on $C_{5}$ is simply transitive, so $C_{5}$ carries its uniform invariant measure. Conditioning on $C_{5}^{\times }$ leaves each of its 119 nonidentity alignments with weight 1/119. Taking the product with the uniform $Z_{3}$ sector therefore assigns weight 1/[3(5!−1)] to each pair.

**Principle** **2** (Single protected ground-state unit)**.** *The primitive confined baryon ground state selects one non-facing unit from the uniformly weighted exposure capacity Equation* (80) *and removes it from the scalar-mass read-out. Its weight is*

(81) $$w_{0}=\frac{1}{3(5!-1)}=\frac{1}{357}.$$

**Remark** **5.** *Conditional on the five-role model, rooted ordering, and the three completed $Z_{3}$ sectors, the chamber capacity is 357. The choice of these five roles, the sign, and the single-unit subtraction in the mass functional remain physical reconstruction postulates rather than consequences of projective topology alone. The identity permutation removed in Equation* (79) *is the trivial embedding; the ground-state unit in Equation* (81) *is a normalized protected state of the resulting confined exposure capacity, not a second removal of the same permutation.*

The leading alpha-free saturated-centroid law is therefore

(82) $$M_{B}^{\left(0\right)}=R^{2}\left(1-\frac{1}{357}+\frac{C_{B}}{64}\right),$$

where $C_{B}$ is a nonnegative leading exposure count relative to the ground pocket.

Define

(83) $$M_{0}=R^{2}\left(1-\frac{1}{357}\right),\;Q_{B}=\frac{R^{2}}{64},$$

so that

(84) $$M_{B}^{\left(0\right)}=M_{0}+C_{B}Q_{B}.$$

## 11. Signed Exposed-Quotient Calculus

A chamber count is not simply the number of formal possibilities. Pure representative redundancies are removed first. The surviving physically distinct exposed incidences may form symmetry orbits with a common sign; the quotient groups such incidences for bookkeeping, while the orbit cardinality retains their physical degeneracy. Protected content is removed from direct read-out, opposite orientations cancel, and the remaining contribution may be positive or negative relative to a lower-level baseline. This section packages those operations into one reusable definition.

The higher corrections must be defined independently of experimental masses. Let

(85) $$D_{n}\left(B\right)=\left(C_{n}\left(B\right),G_{n},P_{n}\left(B\right),\iota_{n},\sigma_{n}\right)$$

be the finite reconstruction datum at interface level *n*, where

- $C_{n}\left(B\right)$ is the chamber set;
- $G_{n}$ is the closure-preserving symmetry group acting on the already IRSP-reduced exposed incidences;
- $P_{n}\left(B\right)$ is protected identity and continuation content;
- $\iota_{n}$ is an orientation-pairing involution;
- $\sigma_{n}:C_{n}\left(B\right)\to \left\{-1,0,+1\right\}$ is the $G_{n}$-invariant carrier-facing signed residue.

**Definition** **3** (Exposed quotient)**.** *The exposed quotient is*

(86) $${\bar{E}}_{n}\left(B\right)=\left(C_{n}\left(B\right)\setminus P_{n}\left(B\right)\right)/G_{n}.$$

*Its signed closure residue is*

(87) $$C_{\mathrm{res}}^{\left(n\right)}\left(B\right)=\sum_{\left[c\right]\in {\bar{E}}_{n}\left(B\right)}m\left(\left[c\right]\right)\;\sigma_{n}\left(\left[c\right]\right),$$

*where m([c])=|[c]| is the number of physically distinct exposed incidences grouped in the orbit. It is a degeneracy, not a reintroduction of representative redundancy: true redundancies have already been removed when constructing $C_{n}\left(B\right)$.*

**Lemma** **5** (Pair cancellation)**.** *If*

(88) $$\iota_{n}^{2}=\mathrm{id},\;\sigma_{n}\left(\iota_{n}c\right)=-\sigma_{n}\left(c\right),$$

*then every non-fixed $\iota_{n}$-pair cancels from Equation* (87)*. Only unpaired, fixed, or asymmetrically weighted residue classes survive.*

**Proof.**  For every two-element orbit $\left\{c,\iota_{n}c\right\}$, the contribution is $m\left(c\right)[\sigma_{n}\left(c\right)+\sigma_{n}\left(\iota_{n}c\right)]=0$ when multiplicities agree. The stated survivors are precisely the cases not removed by this cancellation. □

The familiar ledger

(89) $$C_{\mathrm{res}}^{\left(n\right)}=E_{n}-P_{n}-S_{n}$$

is therefore a compressed notation for Equation (87): positive unpaired exposure contributes +1, protected or paired content contributes zero after quotienting, and over-closure or compensating return contributes −1 relative to the selected baseline.

## 12. Refinement Denominators and Tower Convergence

The leading chamber selects the gross baryon placement. Higher interfaces do not restart the count; they refine an already selected closure at successively smaller scales. This conditional character explains both the product denominator and the rapid convergence of the tower.

The first correction acts on both confined sides and sees both the inherited three-grade structure and the first four-role exposure environment. Let $A_{4}$ denote the four-role exposure set at this interface, with $|A_{4}|=4$, and define

(90) $$F_{4}^{\left(\pm \right)}=G⊔A_{4},\;\left|F_{4}^{\left(\pm \right)}\right|=3+4=7.$$

The two-side boundary family is

(91) $$F_{4}=\left\{-,+\right\}\times F_{4}^{\left(\pm \right)}.$$

**Theorem** **5** (First bilateral refinement denominator)**.** *Under the preceding decomposition, if the first-refinement read-out saturates the fourteen admitted boundary roles with equal weight, then*

(92) $$|F_{4}|=2\left(3+4\right)=14.$$

*Consequently, the first signed-residue quantum is*

(93) $$Q_{B}^{\left(4\right)}=\frac{R^{2}}{64\cdot 14}.$$

After this first bilateral closure has been selected, deeper interfaces refine the already completed orientation class. They do not reopen two independent sides.

**Principle** **3** (One-sided conditional refinement)**.** *At levels n≥5, inverse orientations are closure partners of one completed state. The independent refinement space is the quotient by orientation pairing, contains n roles, and saturates those roles with equal weight. Hence,*

(94) $$D_{n}=n,\;n\geq 5.$$

The full saturated centroid expression is

(95) $$M_{B,\mathrm{cent}}^{\mathrm{sat}}=R^{2}\left[1-\frac{1}{357}+\frac{C_{B}}{64}+\sum_{n=4}^{\infty }\frac{C_{\mathrm{res}}^{\left(n\right)}\left(B\right)}{64D_{4}D_{5}\cdots D_{n}}\right],$$

with $D_{4}=14$ and $D_{n}=n$ for n≥5. In particular,

(96) $$C_{\mathrm{res}}^{\left(4\right)}=\kappa_{B},\;C_{\mathrm{res}}^{\left(5\right)}=\lambda_{B},\;C_{\mathrm{res}}^{\left(6\right)}=\mu_{B}.$$

**Theorem** **6** (Absolute convergence)**.** *If the signed residues satisfy a polynomial bound*

(97) $$|C_{\mathrm{res}}^{\left(n\right)}\left(B\right)|\leq Kn^{p}$$

*for fixed K>0 and p≥0, then the tower in Equation* (95) *converges absolutely.*

**Proof.**  For n≥5,

(98) $$D_{5}D_{6}\cdots D_{n}=5\cdot 6\cdots n=\frac{n!}{4!}.$$

Therefore, the absolute tail is bounded by a constant multiple of

(99) $$\sum_{n=5}^{\infty }\frac{n^{p}}{n!},$$

which converges. □

**Figure 6:**
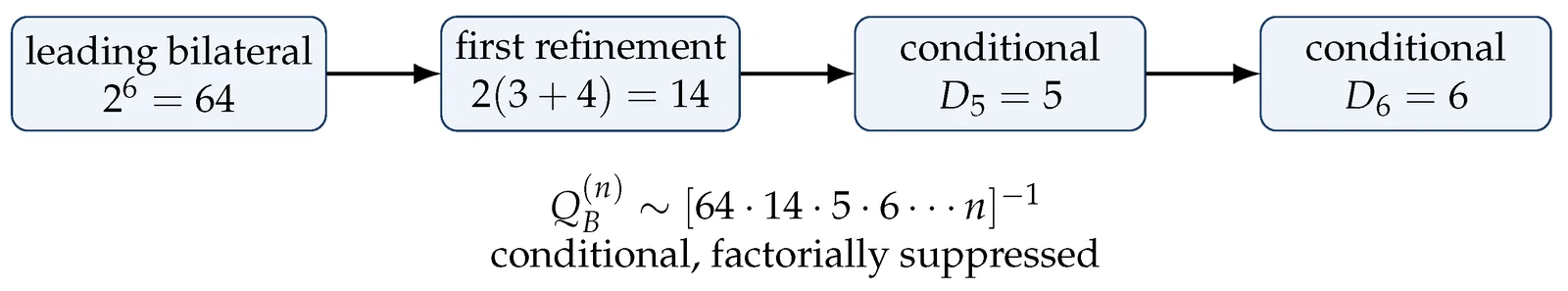
Tower structure. The first refinement is bilateral; later interfaces refine one already selected orientation quotient and therefore contribute one-sided conditional factors.

Figure 6 shows the distinction between the first bilateral refinement and the later one-sided conditional refinements.

## 13. Finite Leading Octet and Decuplet Exposure Operator

The denominator structure is common to all baryons, whereas the family placement is a spectral read-out on the canonical route space $V_{B}=V_{10}⊕V_{8}$. Let $\Pi_{10}$ and $\Pi_{8}$ be the Young-sector projectors. For incidence role *i*, let $S_{i}$ project onto the terminal, or strange-read-out, grade and define

$$S_{i}^{2}=S_{i},\;\left[S_{i},S_{j}\right]=0,\;N_{s}=S_{1}+S_{2}+S_{3}.$$

Inside $V_{8}$, write $P_{n,I}$ for the simultaneous spectral projector onto strange occupancy $N_{s}=n$ and light isospin *I*.

The physical role of these projectors is simple. $N_{s}$ measures how many of the three route roles terminate in the strange-read-out grade, whereas $P_{n,I}$ distinguishes different closure geometries with the same strange occupancy. Thus, Λ and Σ, for example, need not receive the same exposure merely because both have $N_{s}=1$: their singlet and triplet boundary organizations are different. The operator below is a compact, sector-wide rule for these distinctions rather than a separate coefficient assignment for each baryon.

**Structural Postulate** **3** (Leading mass-exposure fibers)**.** *The mixed sector exposes an additive strange-continuation core 3(5−1)=12, a five-role isotriplet boundary when $\left(N_{s},I\right)=\left(1,1\right)$, and a bilateral endcap of weight two when $\left(N_{s},I\right)=\left(2,\frac{1}{2}\right)$. The symmetric sector exposes an aligned bilateral base $2(3^{2}+1)=20$ and an increment 2·5=10 for each strange endpoint.*

**Definition** **4** (Leading exposure operator)**.** *On $V_{B}$, define*

(100) $$\begin{array}{cc} C= & \Pi_{10}\left(20+10N_{s}\right)\Pi_{10}\\ \; & +\Pi_{8}\left(12N_{s}+5P_{1,1}+2P_{2,1/2}\right)\Pi_{8}. \end{array}$$

**Theorem** **7** (Conditional leading-spectrum theorem)**.** *Under Structural Postulate 3, the eigenvalues of C on*

$$\left(N,\Lambda ,\Sigma ,\Xi ;\Delta ,\Sigma^{*},\Xi^{*},\Omega \right)$$

*are*

(101) (0,12,17,26;20,30,40,50).

**Proof.**  In the decuplet, $N_{s}=0,1,2,3$, so the first block of Equation (100) gives 20,30,40,50. In the octet, the nucleon has $N_{s}=0$; the Lambda has $\left(N_{s},I\right)=\left(1,0\right)$; the Sigma has (1,1); and the Xi has $\left(2,\frac{1}{2}\right)$. The second block therefore gives 0,12,12+5,24+2, respectively. □

Table 4 records the same spectrum together with its finite exposure interpretation.

**Remark** **6** (Conditional rather than fitted)**.** *Once Structural Postulate 3 is fixed, no family coefficient is adjusted individually: all eight are eigenvalues of one operator. The remaining physical question is whether a complete premetric carrier calculus selects these leading fibers. The theorem is therefore exact conditionally, but the postulate is not derived from projective topology alone. Nor should it be expected to follow from QCD alone, because QCD begins with the post-read-out baryon degrees of freedom whose prior selection is at issue.*

## 14. Finite Signed Exposure Complex and Centroid-Residue Theorem

The exposed-quotient calculus in Section 11 can now be made finite and explicit. A signed finite set is a pair $X=(X^{+},X^{-})$ with

$$\mathrm{ind}(X)=|X^{+}|-|X^{-}|.$$

Automorphism-related exposed elements carry equal weight because there is no premetric invariant that can distinguish them.

The construction is an incidence version of inclusion–exclusion. Carrier-facing single exposures contribute positively, shared overlaps are removed to avoid double counting, and an overclosed return can contribute negatively relative to the previously selected baseline. The signed index therefore measures a net exposed boundary burden, not a literal number of material constituents.

Let $A_{0}$ be the singleton aligned root, $A_{5}$ a labeled copy of the five-role rooted read-out set $A_{5}$, G the three-grade projective set, and

$$F_{7}=G⊔A_{4},\;O_{6}=\left\{-,+\right\}\times G.$$

Thus,

$$|A_{0}|=1,\;|A_{5}|=5,\;|G|=3,\;|F_{7}|=7,\;|O_{6}|=6.$$

**Definition** **5** (Rooted local exposure complex)**.** *For a symmetric-sector terminal set S⊆{1,2,3}, define*

(102) $$\begin{array}{c} E_{10}^{+}\left(S\right)=\underset{i\in S}{∐}A_{5,i}, \end{array}$$

(103) $$\begin{array}{c} E_{10}^{-}\left(S\right)=A_{0}⊔\underset{\{i,j\}\subseteq S}{∐}G_{ij}⊔\underset{\{1,2,3\}\subseteq S}{∐}F_{7}. \end{array}$$

*For a mixed-sector occupancy vector $x=\left(x_{1},x_{2},x_{3}\right)\in {\left\{0,1\right\}}^{3}$, with $x_{3}$ the continuation role, define*

(104) $$\begin{array}{c} E_{8}^{+}\left(x\right)=x_{3}O_{6}, \end{array}$$

(105) $$\begin{array}{c} E_{8}^{-}\left(x\right)=x_{1}x_{2}O_{6}⊔x_{3}x_{1}G_{31}⊔x_{3}x_{2}G_{32}. \end{array}$$

The aligned root is protected, pair intersections are duplicate grade overlaps, the terminal triple is overclosed, and a side-only mixed singleton is protected by confined-side closure.

For the symmetric sector, the constant, singleton, pair, and triple terms are respectively the aligned root, the five-role terminal exposures, the three-grade overlaps, and the seven-role overclosure. For the mixed sector, the continuation role is the only positive singleton exposure; a double-side occupation and continuation–side overlaps subtract because they close or duplicate an already counted boundary. These interpretations explain the signs before the algebraic index is evaluated.

**Structural Postulate** **4** (Next-grade protected selectors)**.** *At the $Z_{5}$ refinement, the mixed-sector strange singlet $\left(N_{s},I\right)=\left(1,0\right)$ exposes the aligned root once with positive index. At the $Z_{6}$ refinement, the mixed-sector strange isotriplet $\left(N_{s},I\right)=\left(1,1\right)$ exposes the two compensating orientation ends with negative index. All other octet and decuplet classes at these two grades are protected or paired. Equivalently, the signed fibers have indices*

$$\mathrm{ind}E_{5}=P_{1,0},\;\mathrm{ind}E_{6}=-2P_{1,1}.$$

**Theorem** **8** (Exposure-index and no-extra-motif theorem)**.** *The signed indices are*

(106) indE10(S)=−1+5|S|−3|S|2−7|S|3,

(107) $$\begin{array}{c} \mathrm{ind}E_{8}\left(x\right)=6x_{3}-6x_{1}x_{2}-3x_{3}\left(x_{1}+x_{2}\right). \end{array}$$

*Among grade-local multilinear valuations with the declared protection, side exchange, and Young-sector support, these complexes exhaust all nonvanishing first-refinement motif types.*

**Proof.**  Additivity of the signed index immediately gives Equations (106) and (107). For completeness, a symmetric multilinear function of three binary occupancies is spanned by

1,Ns,Ns2,Ns3,

which are precisely the root, singleton, pair, and triple motifs above. In the side-symmetric mixed sector, the invariant multilinear monomials are

$$1,\;x_{1}+x_{2},\;x_{3},\;x_{1}x_{2},\;x_{3}\left(x_{1}+x_{2}\right),\;x_{1}x_{2}x_{3}.$$

Protection removes the first two, and the last projects entirely into the symmetric sector. The remaining three are exactly the continuation, double-side, and continuation–side motifs in $E_{8}$. □

The phrase “no extra motif” has a precise finite meaning here. A grade-local rule on three binary occupancies is a multilinear Boolean function, so the monomials listed in the proof form a complete basis. After protection and side symmetry are imposed, there is no unlisted local term that could be added at this refinement order without changing one of the declared premises.

Promoting the occupancy indicators in the finite indices to commuting projectors turns the signed counts into operators on the full 10+8 route space. This is the step that converts a finite combinatorial ledger into a simultaneous mass rule for every family.

**Definition** **6** (Centroid-residue operators)**.** *Define*

(108) K10(4)=−1+5Ns−3Ns2−7Ns3,

(109) $$\begin{array}{c} K_{8}^{\left(4\right)}=6S_{3}-6S_{1}S_{2}-3S_{3}\left(S_{1}+S_{2}\right), \end{array}$$

(110) $$\begin{array}{c} R_{4}=\Pi_{10}K_{10}^{\left(4\right)}\Pi_{10}+\Pi_{8}K_{8}^{\left(4\right)}\Pi_{8}, \end{array}$$

(111) $$\begin{array}{c} R_{5}=P_{1,0},\;R_{6}=-2P_{1,1}. \end{array}$$

**Theorem** **9** (Conditional centroid-residue spectrum)**.** Under Definition 5 and Structural Postulate 4, the spectra of $R_{4},R_{5},R_{6}$ on $\left(N,\Lambda ,\Sigma ,\Xi ;\Delta ,\Sigma^{*},\Xi^{*},\Omega \right)$ are

(112) $$\begin{array}{c} \mathrm{spec}R_{4}=(0,0,4,-3;-1,4,6,-2), \end{array}$$

(113) $$\begin{array}{c} \mathrm{spec}R_{5}=(0,1,0,0;0,0,0,0), \end{array}$$

(114) $$\begin{array}{c} \mathrm{spec}R_{6}=(0,0,-2,0;0,0,0,0). \end{array}$$

**Proof.**  In the symmetric sector, substitution of $N_{s}=0,1,2,3$ into Equation (108) gives (−1,4,6,−2). In the mixed sector, evaluation of Equation (109) on normalized side-symmetric representatives is equally explicit. The *N* and Λ representatives give zero. For Σ, the normalized mixed-symmetry vector has squared weight 2/3 on the continuation-strange configuration, where $K_{8}^{\left(4\right)}=6$, and squared weight 1/3 on the side-strange configuration, where $K_{8}^{\left(4\right)}=0$; its eigenvalue is therefore 4. For Ξ, the corresponding squared weights are 2/3 on the two-side-strange configuration, where $K_{8}^{\left(4\right)}=-6$, and 1/3 on the side-plus-continuation configuration, where $K_{8}^{\left(4\right)}=3$; its eigenvalue is −3. These are the $S_{\left(2,1\right)}$ components orthogonal to the fully symmetric state, giving (0,0,4,−3). The last two spectra follow directly from the intrinsic $\left(N_{s},I\right)$ projectors and Structural Postulate 4. The motif classification in Theorem 8 proves that no additional first-refinement term exists inside the declared local class. □

Writing the three spectra as $\kappa_{B},\lambda_{B},\mu_{B}$, respectively, gives

(115) $$\begin{array}{ccccccccc} B & N & \Lambda & \Sigma & \Xi & \Delta & \Sigma^{*} & \Xi^{*} & \Omega \\ C_{B} & 0 & 12 & 17 & 26 & 20 & 30 & 40 & 50\\ \kappa_{B} & 0 & 0 & 4 & -3 & -1 & 4 & 6 & -2\\ \lambda_{B} & 0 & 1 & 0 & 0 & 0 & 0 & 0 & 0\\ \mu_{B} & 0 & 0 & -2 & 0 & 0 & 0 & 0 & 0 \end{array}$$

and the complete displayed centroid operator is

(116) $${\hat{M}}_{\mathrm{cent}}^{\left(6\right)}=M_{0}1+Q_{B}C+Q_{B}^{\left(4\right)}R_{4}+Q_{B}^{\left(5\right)}R_{5}+Q_{B}^{\left(6\right)}R_{6}.$$

**Remark** **7** (Logical and historical status)**.** *The spectra in Equation* (114) *now follow formally from one finite exposure complex; the baryons are not assigned eight unrelated correction integers. The small ledger historically helped identify the relevant fibers, so its agreement with the same masses is not a blind prediction. The valid claim is a conditional finite reconstruction whose remaining physical premise is that a complete premetric carrier calculus selects Definition 5 and Structural Postulate 4.*

## 15. Centroid-Preserving Orientation Residues

Members of one charge multiplet may carry different intrinsic boundary orientations, but a pure orientation splitting cannot change the multiplet average. The calculation therefore separates into two questions. First, which vectors redistribute mass while preserving the centroid? Second, which finite carrier-facing source generates the amplitudes of those vectors? The first question is representation-theoretic; the second is the substantive reconstruction problem addressed by the carrier-lift complex below. Let $A_{B}$ be the charge-orientation orbit and let $c_{n}:A_{B}\to Z$ be a level-*n* orientation residue. Its singlet projection is

(117) $$\Pi_{0}c_{n}=\frac{1}{|A_{B}|}\sum_{a\in A_{B}}c_{n}\left(a\right).$$

**Theorem** **10** (Centroid-preservation theorem)**.** *A pure orientation splitting contains no singlet component if and only if*

(118) $$\sum_{a\in A_{B}}c_{n}\left(a\right)=0.$$

*Thus, every trace-free orientation residue preserves the multiplet centroid.*

**Proof.**  The centroid is the constant or trivial-representation component of a function on the orientation orbit. Removing that component leaves the subspace orthogonal to constants, which is exactly the zero-sum subspace. □

For an isotriplet the trace-free space is spanned by $I_{3}$ and $3I_{3}^{2}-2$; for a doublet, it is one-dimensional. The amplitudes are fixed below by converting the problem into an incidence boundary.

### 15.1. Isospin Paths and Unique Edge Flows

An isospin multiplet can be viewed as a one-dimensional chain of adjacent charge states. An edge flow records the signed structural transfer between neighboring weights, and its boundary records what each state gains or loses. This converts a zero-sum splitting vector into the discrete analogue of a conserved current with sources at the vertices.

Order an isospin multiplet by descending $I_{3}$ and let its *d* weights form the path $v_{1}-\cdots -v_{d}$. Orient each edge from $v_{j}$ to $v_{j+1}$. For an edge flow $f=(f_{1},\ldots ,f_{d-1})$, define

(119) $$\partial_{I}f=\left(f_{1},-f_{1}+f_{2},\ldots ,-f_{d-2}+f_{d-1},-f_{d-1}\right).$$

**Proposition** **3** (Integral boundary representation)**.** *Every integer zero-sum charge vector is the boundary of a unique integer edge flow.*

**Proof.**  For $c=(c_{1},\ldots ,c_{d})$ with $\sum_{j}c_{j}=0$, set

$$f_{j}=\sum_{k=1}^{j}c_{k}.$$

Then, $\partial_{I}f=c$. A path has no nonzero cycle flow, so the representation is unique. □

For a triplet $c=(c_{+},c_{0},c_{-})$, the unique flow is simply $\left(c_{+},c_{+}+c_{0}\right)$. Thus, the combined Sigma vector (−108,−15,123) becomes the two-edge flow (−108,−123). The flow contains the same information as the charge vector, but it makes the oriented source–return structure visible.

The historically identified level columns therefore reduce to

(120) $$\begin{array}{cccc} \; & n=4 & n=5 & n=6\\ \Sigma & \left(-4,-4\right) & \left(2,0\right) & \left(0,-3\right)\\ \Xi & \left(-3\right) & \left(-1\right) & \left(-2\right)\\ \Sigma^{*} & \left(-2,-3\right) & \left(1,2\right) & \left(4,3\right)\\ \Xi^{*} & \left(-1\right) & \left(-2\right) & (-3). \end{array}$$

### 15.2. Why Sector Conditioning Is Necessary

The next test asks whether one universal polynomial in the three light-flavor labels could generate all observed octet and decuplet edge flows. If it could, no explicit 8/10 sector distinction would be needed. The null relations below show that this simpler possibility is algebraically too restrictive.

Let τ(u)=1,τ(d)=−1,τ(s)=0, with $\tau_{i}$ acting on role *i*. The most general diagonal role-local quadratic expression invariant under side exchange is

(121) $$\begin{array}{cc} X_{2}= & a\left(\tau_{1}+\tau_{2}\right)+b\tau_{3}+c\tau_{1}\tau_{2}+d\tau_{3}\left(\tau_{1}+\tau_{2}\right)\\ \; & +e\left(\tau_{1}^{2}+\tau_{2}^{2}\right)+f\tau_{3}^{2}. \end{array}$$

**Theorem** **11** (Quadratic-local obstruction)**.** *If the centered path-flow modes are ordered as*

$$(f_{\Sigma ,1},f_{\Sigma ,2},f_{\Xi },f_{\Sigma^{*},1},f_{\Sigma^{*},2},f_{\Xi^{*}}),$$

*every sector-blind selector of the form Equation* (121) *satisfies*

(122) $$\begin{array}{cc} 3\left(f_{\Sigma^{*},1}+f_{\Sigma^{*},2}\right)-2\left(f_{\Sigma ,1}+f_{\Sigma ,2}\right)-4f_{\Xi } & =0, \end{array}$$

(123) $$\begin{array}{cc} 6f_{\Xi^{*}}-\left(f_{\Sigma ,1}+f_{\Sigma ,2}\right)-2f_{\Xi } & =0. \end{array}$$

*The three columns of Equation* (120) *violate these relations by (13,8), (9,−12), and (35,−11), respectively.*

**Proof.**  Evaluation of the six monomials in Equation (121) on the exact octet and decuplet flavor weights gives a mode matrix of rank four. An integer basis for its two left-null relations is precisely Equations (122) and (123). Direct substitution gives the displayed nonzero pairs. □

Thus, a genuine octet/decuplet-conditioned carrier exposure is required; a sector-blind polynomial in charge labels cannot derive the ledger.

### 15.3. Combined Normal Form

The individual level-four, level-five, and level-six orientation columns are not separately observable at this truncation: their mass quanta differ by the fixed factors 30 and 6. Combining them removes this decomposition ambiguity and isolates the single charge operator that the mass spectrum actually tests.

Because

$$Q_{B}^{\left(4\right)}=30Q_{B}^{\left(6\right)},\;Q_{B}^{\left(5\right)}=6Q_{B}^{\left(6\right)},$$

the observable through level six depends on the combined orientation operator

$$O_{\leq 6}=30O_{4}+6O_{5}+O_{6}.$$

The resolved non-nucleon vectors and flows combine to

(124) $$\begin{array}{ccc} B & K_{B,a}^{\left(6\right)} & h_{B}\\ \Sigma & \left(-108,-15,123\right) & \left(-108,-123\right)\\ \Xi & \left(-98,98\right) & \left(-98\right)\\ \Sigma^{*} & \left(-50,-25,75\right) & \left(-50,-75\right)\\ \Xi^{*} & \left(-45,45\right) & \left(-45\right) \end{array}.$$

Let

$$T_{2}=3I_{3}^{2}-I\left(I+1\right)1.$$

**Proposition** **4** (Unique combined normal form)**.** *Suppose the dipole coefficient is affine in $N_{s}$ within each Young sector, the quadrupole coefficient is $N_{s}$-independent within that sector, and both sectors share the same unstranged carrier-root coefficient. Then, Equation* (124) *determines uniquely*

(125) $$\begin{array}{cc} O_{\leq 6}= & \Pi_{8}\left[\left(-35-\frac{161}{2}N_{s}\right)I_{3}+\frac{15}{2}T_{2}\right]\Pi_{8}\\ \; & +\Pi_{10}\left[\left(-35-\frac{55}{2}N_{s}\right)I_{3}+\frac{25}{2}T_{2}\right]\Pi_{10}. \end{array}$$

**Proof.**  For a triplet, a vector $AI_{3}+B\left(3I_{3}^{2}-2\right)$ has path flow (A+B,A−B); a doublet has $T_{2}=0$ and flow A/2. The octet data give $A_{8}\left(1\right)=-231/2$, $A_{8}\left(2\right)=-196$, $B_{8}=15/2$; the decuplet gives $A_{10}\left(1\right)=-125/2$, $A_{10}\left(2\right)=-90$, $B_{10}=25/2$. Affine continuation gives the same unstranged coefficient −35 in both sectors, which fixes Equation (125). □

The logical order is important. The six independent observed edge-flow components in Equation (124) determine the five coefficients in Equation (125) subject to the shared-root and affine-continuation restrictions. Their subsequent factorization into small cardinalities does not turn those five coefficients into blind predictions. The independently checkable content at this stage is the cross-multiplet restriction, the obstruction to the simpler sector-blind selector in Theorem 11, and the prospective extension to the unmeasured Delta vector after the carrier fibers are frozen.

### 15.4. Saturated Carrier-Lift Complex

This is the most model-dependent step of the charge reconstruction. It gives a finite carrier-fiber realization of the five coefficients in Equation (125), assembled from role sets already used in the centroid construction. Those fibers were recognized after the coefficients had been extracted from the non-Delta spectrum; they are a retrospective structural reinterpretation unless and until selected by an independent carrier calculus. The intuition is that a charge splitting is not attached directly to a flavor label; it is the boundary left when an oriented isospin path is lifted back through every admitted carrier-facing realization. Saturation then means counting each admitted lift once.

The five coefficients factor only through role sets already present in the centroid construction:

(126) $$35=5\cdot 7,\;161=7\left(5^{2}-2\right),\;55=5\left(3+4+4\right),\;15=3\cdot 5,\;25=5^{2}.$$

Write

$$G=\left\{1,u,u^{2}\right\},\;F_{7}=G⊔A_{4},$$

and let $A_{4,\mathrm{out}}$ and $A_{4,\mathrm{ret}}$ be two labeled copies of $A_{4}$. Also let

$$P=\left\{\left(\varepsilon ,\varepsilon \right),\left(\eta ,\eta \right)\right\}\subset A_{5}\times A_{5}.$$

**Definition** **7** (Carrier-lift fibers)**.** *Define*

(127) $$\begin{array}{c} D_{0}=A_{5}\times F_{7}, \end{array}$$

(128) $$\begin{array}{c} D_{s,8}=F_{7}\times \left((A_{5}\times A_{5})\setminus P\right), \end{array}$$

(129) $$\begin{array}{c} D_{s,10}=A_{5}\times \left(G⊔A_{4,\mathrm{out}}⊔A_{4,\mathrm{ret}}\right), \end{array}$$

(130) $$\begin{array}{c} Q_{8}=G\times A_{5},\;Q_{10}=A_{5}\times A_{5}. \end{array}$$

**Structural Postulate** **5** (Carrier-lift saturation)**.** *After the primitive return orientation is selected, every rooted route in Definition 7 is admitted once and all such routes are saturated. The mixed sector retains an ordered outgoing–return pair and protects the two nonprojective diagonals P. The symmetric sector identifies the aligned pair but retains outgoing and returning four-face incidences. A marked strange grade is additive on the oriented edge, while endpoint deficiency is a joint bilateral mode whose read-out is shared equally by the two confined sides.*

The resulting fiber cardinalities are

(131) $$\begin{array}{cccccc} X & D_{0} & D_{s,8} & D_{s,10} & Q_{8} & Q_{10}\\ \left|X\right| & 35 & 161 & 55 & 15 & 25 \end{array}.$$

The numbers have direct bookkeeping meanings. $D_{0}$ is the common rooted source, $D_{s,8}$ and $D_{s,10}$ are the strange-grade lifts in the mixed and symmetric sectors, and $Q_{8},Q_{10}$ are the corresponding endpoint-deficiency fibers. Their unequal sizes are why the no-go result in Theorem 11 requires octet/decuplet conditioning.

A useful physical analogy is a conserved flow through a small network. The vertices are the observable charge states and the edges record how much signed boundary burden is transferred between neighboring states. Counting all admissible carrier lifts fixes the strength of that flow; taking its boundary tells which charge state gains or loses mass. Because every internal transfer leaves one vertex and enters another, the total over a multiplet remains zero and its centroid is unchanged.

For a multiplet of dimension d=2I+1, define on its *k*-th edge

(132) $$u_{k}=\frac{k(d-k)}{2},\;v_{k}=\frac{k(d-k)(d-2k)}{2}.$$

**Lemma** **6** (Canonical isospin edge primitives)**.**

$$\partial_{I}u=I_{3},\;\partial_{I}v=T_{2}.$$

*Moreover, $2u_{k}=k\left(d-k\right)$ is the squared SU(2) lowering coefficient on the k-th weight edge.*

**Proof.**  The inverse of $\partial_{I}$ is cumulative summation on zero-sum vertex vectors. Hence,

$$\sum_{j=0}^{k-1}\left(I-j\right)=\frac{k(d-k)}{2},$$

and summing $3{\left(I-j\right)}^{2}-I\left(I+1\right)$ gives the stated $v_{k}$. The usual lowering coefficient from $I_{3}=I-k+1$ is k(d−k). □

The mode *u* is the discrete dipole primitive and *v* is its trace-free quadratic companion. Taking their boundary produces the familiar $I_{3}$ and quadrupole patterns on the charge states; multiplying these universal shapes by the saturated fiber indices supplies the source strengths.

**Theorem** **12** (Conditional carrier-source cochain)**.** *Under Structural Postulate 5, the saturated source cochains are*

(133) $$\begin{array}{cc} h_{8}\left(N_{s}\right) & =-\left(35+\frac{161}{2}N_{s}\right)u+\frac{15}{2}v, \end{array}$$

(134) $$\begin{array}{cc} h_{10}\left(N_{s}\right) & =-\left(35+\frac{55}{2}N_{s}\right)u+\frac{25}{2}v. \end{array}$$

*Their boundaries equal Equation* (125)*; on all non-nucleon light multiplets, they are integral and give*

(135) $$\begin{array}{cccccc} B & \Sigma & \Xi & \Delta & \Sigma^{*} & \Xi^{*}\\ h_{B} & \left(-108,-123\right) & \left(-98\right) & \left(-15,-70,-90\right) & \left(-50,-75\right) & \left(-45\right) \end{array}.$$

**Proof.**  The primitive route contributes $-|D_{0}|u=-35u$. The strange and endpoint modes are bilateral joint modes and carry one half of the corresponding fiber index. Substitution of Equation (131) gives Equation (134); Lemma 6 then gives the boundary operator. Direct evaluation gives Equation (135). □

**Corollary** **2** (Conditional Delta charge vector)**.** *In the order $\left(\Delta^{++},\Delta^{+},\Delta^{0},\Delta^{-}\right)$,*

(136) $$K_{\Delta ,a}^{\left(6\right)}=\left(-15,-55,-20,90\right).$$

**Proof.**  For $N_{s}=0$, I=3/2, the canonical modes give $I_{3}=\left(3/2,1/2,-1/2,-3/2\right)$ and $T_{2}=\left(3,-3,-3,3\right)$. Substitution into the decuplet block of Equation (125) gives the result. □

The displayed level-six mass formula is therefore

(137) $$M_{B,a}^{\left(6\right)}=\langle B|{\hat{M}}_{\mathrm{cent}}^{\left(6\right)}|B\rangle +K_{B,a}^{\left(6\right)}Q_{B}^{\left(6\right)}.$$

The proton and neutron use the more resolved dedicated ground-state closures in Section 17. Formally extending the common source to the nucleon doublet gives $m_{p}-m_{n}=-35Q_{B}^{\left(6\right)}=-1.226001560$ MeV, whereas the dedicated closure gives −1.293332153 MeV. The difference isolates an additional ground-state closure contribution rather than an adjustable correction.

**Remark** **8** (Exact theorem, explicit physical premise)**.** *The finite boundary calculation is exact under Structural Postulate 5. Saturation includes every admitted route but does not itself determine which incidence fibers nature realizes. The carrier-lift postulate is therefore the precise physical correspondence premise of the charge derivation; it replaces an unstructured list of charge integers by one testable finite map.*

## 16. Resolved Nucleon Ground-State Closures

The common octet operator assigns the nucleon family the coarse centroid coefficient $C_{N}=0$. Resolving the two members of the isospin doublet requires additional ground-state information: the neutron is modeled as the lowest closed-neutral realization of the bilateral parent, whereas the proton is modeled as the lowest charged-boundary opening of the same pocket. These are not two unrelated mass formulas; they are two boundary conditions on the common neutral-parent baryon closure.

### 16.1. Binary Projective Refinement of the Three-Grade Carrier

The three projective grades used in the primitive channel theorem can also support a finer internal overlap question. Retain their binary incidence content and form

(138) $$W_{2}:=F_{2}\langle g_{0},g_{1},g_{2}\rangle ,\;P_{2}:=W_{2}\setminus \left\{0\right\}.$$

The seven nonzero vectors are the points of the finite projective plane PG(2,2), usually called the Fano plane. Because $F_{2}$ has only one nonzero scalar, no further rescaling quotient is required. A projective line is the set of three nonzero vectors in a two-dimensional subspace:

(139) $$L_{2}:=\left\{\;U\setminus \left\{0\right\}:U\leq W_{2},\;\dim_{F_{2}}U=2\;\right\}.$$

The use of the binary span at this refinement level is explicit and conditional. It does not assert that every element of GL(3,2) preserves the degree or multiplication in the graded cohomology ring $F_{2}\left[u\right]/\left(u^{3}\right)$. The primitive channel theorem continues to distinguish $g_{0},g_{1},g_{2}$. The additional premise used here is only that their first internal *overlap* read-out retains the nonzero binary incidence pattern but is insensitive to the names of the three basis representatives.

This finite plane is not a second spacetime and its points are not new particles. It is an incidence diagram for internal binary co-activations of the three already established grades. In particular, a line has the closure form {a,b,a+b}: any two of its entries determine the third. This makes a line the natural finite representative of a closed three-sheet support, while the four points outside it represent the first open four-slot complement.

**Definition** **8** (Nucleon binary-incidence fibers)**.** *Fix a closed-support line $l\in L_{2}$, and define*

(140) $$\begin{array}{c} P_{5}:=\{A\subseteq \{1,\ldots ,5\}:|A|=2\}, \end{array}$$

(141) $$\begin{array}{c} N_{l}:=P_{5}\times l, \end{array}$$

(142) $$\begin{array}{c} \Phi :=\{\left(l^{\prime },x\right):l^{\prime }\in L_{2},\;x\in l^{\prime }\}, \end{array}$$

(143) $$\begin{array}{c} \Phi^{\pm }:=\{-,+\}\times \Phi , \end{array}$$

(144) $$\begin{array}{c} H_{l}:=\{A\in GL\left(W_{2}\right):Al=l\}. \end{array}$$

*Here, $N_{l}$ is the neutral pair fiber marked by a closed-sheet position,* Φ *is the point–line incidence or flag fiber, $\Phi^{\pm }$ adds the two charged-boundary orientations, and $H_{l}$ is the group of binary relabelings that preserves the selected closed support.*

**Lemma** **7** (Fano incidence ledger)**.** *The fibers in Definition 8 satisfy*

(145) $$\begin{array}{c} |P_{2}\left|=7,\;\right|L_{2}\left|=7,\;|l|=3,\;\right|P_{2}\setminus l|=4,\\ |N_{l}\left|=30,\;|\Phi |=21,\;\right|\Phi^{\pm }|=42,\\ H_{l}≅S_{4},\;\left|H_{l}\setminus \left\{\mathrm{id}\right\}\right|=23. \end{array}$$

**Proof.**  The three-dimensional binary vector space has $2^{3}-1=7$ nonzero vectors. A two-dimensional subspace has $2^{2}-1=3$ nonzero vectors, so its complement in $P_{2}$ has four. There are 7·6 ordered independent pairs in $W_{2}$; each two-dimensional subspace has 3·2 ordered bases. Hence, there are (7·6)/(3·2)=7 lines and 7·3=21 flags. Adding two boundary orientations gives 42, while |Nl|=52·3=30.

Finally,

(146) $$\left|GL\left(3,2\right)\right|=\left(2^{3}-1\right)\left(2^{3}-2\right)\left(2^{3}-2^{2}\right)=168.$$

The group acts transitively on the seven lines, so orbit–stabilizer gives $|H_{l}|=168/7=24$. The four complementary points span $W_{2}$, so an element fixing all four is the identity; hence, the induced action on those points is faithful. Because both $H_{l}$ and their full permutation group have order 24, the image is all of $S_{4}$. Removing the identity leaves 24−1=23 nontrivial line-preserving modes. □

**Figure 7:**
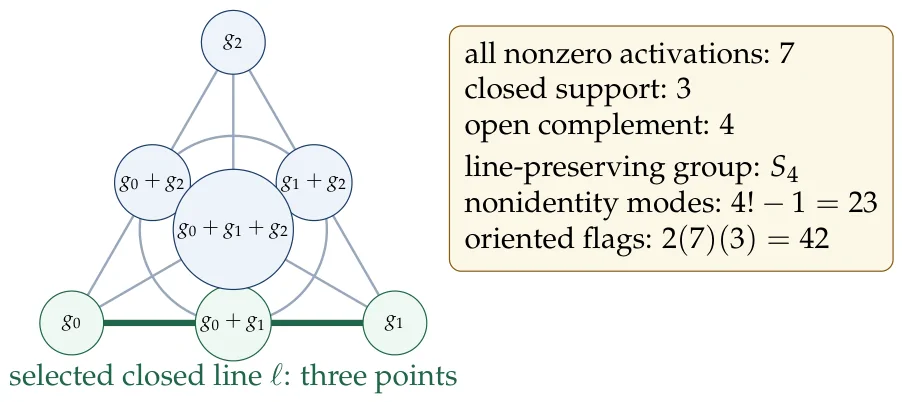
Binary projective refinement used for the nucleon correction indices. The diagram is the Fano plane of the three-grade binary span, not a physical-space picture. Selecting one three-point line as the closed support leaves a four-point complement. The symmetries preserving that line permute the four complementary points as $S_{4}$.

Figure 7 gives a visual reading of this ledger. The green line is the closed three-sheet support; the four blue points form its open complement.

### 16.2. First Exposed Four-Slot Interface

The four-slot interface now has an invariant origin: it is the complement $P_{2}\setminus l$ of the selected closed line. Its line-preserving relabelings form $H_{l}≅S_{4}$. The identity relabeling preserves the already completed return and is protected rather than carrier-facing, leaving

(147) $$a_{4}:=\left|H_{l}\setminus \left\{\mathrm{id}\right\}\right|=4!-1=23.$$

Thus, the same finite geometry produces both the closed three-sheet support and the nontrivial orderings of its four-slot complement.

### 16.3. Closed-Neutral Neutron Count

The closed-neutral read-out selects a paired support inside a five-role environment, giving 52=10 choices. Each paired support sees two opposed four-slot exposures and one protected neutral spine. Its conditional capacity is therefore

(148) Nn=52[2a4+1]=10(47)=470.

The leading subtraction $1/N_{n}$ is the inverse information capacity of this declared exposure space.

### 16.4. Charged-Boundary Proton Count

The charged read-out opens an ordered five-slot boundary while retaining the non-protected four-slot exposure $a_{4}$. Two boundary orientations give

(149) $$N_{p}=2\left[\;5!+\left(4!-1\right)\;\right]=2\left(120+23\right)=286.$$

This form is used consistently: the protected subtraction belongs to the four-slot exposure, not to the total five-plus-four-slot sum.

**Structural Postulate** **6** (Binary projective overlap read-out)**.** *At the first internal refinement of a selected nucleon closure, all nonzero binary activations in $W_{2}$ are saturated with their invariant equal weight, and the closed $Z_{3}$ support reads out as one projective line ℓ. The closed-neutral second-order source is one unmarked paired self-overlap plus the diagonal self-incidence of $N_{l}$, with outer normalization over $P_{5}$. Charged-boundary screening is the incidence of the selected closed line ℓ inside $P_{2}$. The next restoration counts every nonidentity line-preserving mode in $H_{l}$ once and normalizes their total incidence over the oriented flag fiber $\Phi^{\pm }$. Self-overlap and screening reduce the unresolved carrier-facing burden, while a line-preserving return restores protected content after screening.*

This postulate specifies one geometric read-out rule rather than the three fractions themselves. The fractions are consequences of the finite ledger.

**Theorem** **13** (Conditional nucleon correction-index theorem)**.** *Under Structural Postulate 6, the neutral seam-displacement index, charged screening index, and charged restoration index are*

(150) $$d_{n}=\frac{1}{30},\;s_{p}=\frac{3}{7},\;r_{p}=\frac{23}{42}.$$

*Consequently,*

(151) $$\eta_{n}=\frac{1+d_{n}}{|P_{5}|}=\frac{1+1/30}{10},\;\eta_{p}=s_{p}=\frac{3}{7},\;\zeta_{p}=r_{p}=\frac{23}{42}.$$

**Proof.**  Let $\Delta N_{l}=\left\{\left(x,x\right):x\in N_{l}\right\}$ be the diagonal in two copies of the neutral marked-pair fiber. The transitive relabeling action gives equal weight to its 30 elements, so the normalized diagonal incidence is

(152) $$d_{n}=\frac{|\Delta N_{l}|}{|N_{l}\times N_{l}|}=\frac{30}{30^{2}}=\frac{1}{30}.$$

The charged screening support is one three-point line inside the seven-point activation set, giving

(153) $$s_{p}=\frac{\left|l\right|}{|P_{2}|}=\frac{3}{7}.$$

Finally, the restoration source contains the 23 nonidentity elements of $H_{l}$, while the target exposure has 42 oriented flags. The normalized restoration incidence specified in Structural Postulate 6 is therefore

(154) $$r_{p}=\frac{|H_{l}\setminus \left\{\mathrm{id}\right\}|}{|\Phi^{\pm }|}=\frac{23}{42}.$$

Substitution into the neutral pair normalization $|P_{5}{|}^{-1}=1/10$ proves Equation (151). □

The diagonal calculation should not be read as a random event occurring in time. It is the normalized weight of the common part of two equal finite copies, just as the overlap of two identical lists contains one matching entry for each entry in the list. Likewise, 3/7 is the fraction of saturated binary activations lying on the selected closed support, and 23/42 compares closure-preserving rearrangements with all oriented support flags. The negative second-order terms remove repeated or screened exposure; the positive third-order term returns the protected part that the charged screening would otherwise remove twice.

The resulting closure factors are

(155) $$F_{n}=1-\frac{1}{N_{n}}-\frac{\eta_{n}}{N_{n}^{2}}=1-\frac{1}{470}-\frac{1+1/30}{10\cdot 470^{2}},$$

(156) $$\begin{array}{cc} F_{p} & =1-\frac{1}{N_{p}}-\frac{\eta_{p}}{N_{p}^{2}}+\frac{\zeta_{p}}{N_{p}^{3}}\\ \; & =1-\frac{1}{286}-\frac{3}{7\cdot 286^{2}}+\frac{23}{42\cdot 286^{3}}. \end{array}$$

Together with the common rest-mass map Equation (17), these give

(157) $$\frac{m_{n}}{m_{e}}=\frac{R^{2}}{m_{e}}F_{n},\;\frac{m_{p}}{m_{e}}=\frac{R^{2}}{m_{e}}F_{p}.$$

**Remark** **9** (Logical and historical status)**.** *Equations* (148) *and* (149) *are explicit counts inside the declared closed-neutral and charged-boundary exposure spaces, and Theorem 13 and Equations* (155) *and* (156) *now derive the three correction fractions from one finite binary-incidence geometry under Structural Postulate 6. The historical search was guided by the measured nucleon masses, so the numerical agreement is still not advertised as a blind fit-independent prediction. The remaining structural target is narrower: derive the binary projective overlap read-out, including its normalized restoration incidence, from a complete premetric carrier calculus and prove that no inequivalent admissible refinement gives a competing result. This target does not require a conventional spacetime Lagrangian at the premetric level.*

### 16.5. Why Different Counts Still Give Nearly Degenerate Masses

The counts 470 and 286 do not multiply the common scale. They enter through small inverse-capacity deficits:

(158) $$F_{C}=1-O\left(N_{C}^{-1}\right),\;C=n,p.$$

Both nucleons are therefore saturated ground-state read-outs close to the same inherited scale $R^{2}$, and their difference is controlled by the difference between two small boundary residues. This explains within the model why substantially different chamber capacities can nevertheless produce an isospin doublet whose masses agree to three significant figures. It does not replace the QCD + QED account of that splitting into strong-isospin and electromagnetic contributions.

## 17. Numerical Evaluation of the Conditional Reconstruction

The numerical comparison follows the status audit in Table 3. No continuously adjustable baryon-sector scale is introduced: $R^{2}/m_{e}$ is inherited from the charged-lepton construction, and measured $m_{e}$ only converts dimensionless mass ratios to MeV. The leading truncation is shown separately from the complete displayed centroid operator Equation (116). Where a residual is divided by a quoted experimental uncertainty, the resulting quantity is denoted $z_{\exp}$. It is a descriptive normalization only: no theoretical uncertainty has yet been assigned to the reconstruction premises, so $z_{\exp}$ is not a theory pull or a statistical significance. The nucleon ratios are reported in Table 5, the leading and refined non-nucleon centroids in Table 6 and Table 7, and the resolved charge indices, non-Delta comparison, and frozen Delta values in Table A1, Table A2 and Table A3.

Using

(159) $${\left(\frac{m_{\mu }}{m_{e}}\right)}_{\mathrm{str}}=206.768282689,\;{\left(\frac{m_{\tau }}{m_{e}}\right)}_{\mathrm{str}}=3477.441636,$$

from Ref. [11], one obtains

(160) $$\frac{R^{2}}{m_{e}}=1842.6049593445.$$

Using the CODATA electron mass energy equivalent $m_{e}c^{2}=0.51099895069\left(16\right)\;\mathrm{MeV}$, solely as the external reporting unit and with c=1, gives [17]

(161) $$R^{2}=941.5692008\;\mathrm{MeV}.$$

The structural quanta are

(162) $$\begin{array}{c} M_{0}=R^{2}\left(1-\frac{1}{357}\right)=938.9317521\;\mathrm{MeV}, \end{array}$$

(163) $$\begin{array}{c} Q_{B}=R^{2}/64=14.71201876\;\mathrm{MeV}, \end{array}$$

(164) $$\begin{array}{c} Q_{B}^{\left(4\right)}=R^{2}/\left(64\cdot 14\right)=1.050858483\;\mathrm{MeV}, \end{array}$$

(165) $$\begin{array}{c} Q_{B}^{\left(5\right)}=R^{2}/\left(64\cdot 14\cdot 5\right)=0.210171697\;\mathrm{MeV}, \end{array}$$

(166) $$\begin{array}{c} Q_{B}^{\left(6\right)}=R^{2}/\left(64\cdot 14\cdot 5\cdot 6\right)=0.035028616\;\mathrm{MeV}. \end{array}$$

The primary non-nucleon comparison uses only

(167) $$M_{B}^{\left(0\right)}=M_{0}+C_{B}Q_{B}.$$

The displayed conditional centroid reconstruction is

(168) $$M_{B,\mathrm{cent}}^{\left(6\right)}=M_{0}+C_{B}Q_{B}+\kappa_{B}Q_{B}^{\left(4\right)}+\lambda_{B}Q_{B}^{\left(5\right)}+\mu_{B}Q_{B}^{\left(6\right)}.$$

### 17.1. Resolved Proton and Neutron Ratios

The generic $C_{N}=0$ centroid is only a coarse common ground-state baseline. The closed-neutral and charged-boundary resolutions constructed in Section 16 instead give

(169) $$\frac{m_{n}}{m_{e}}=1838.683661321,\;\frac{m_{p}}{m_{e}}=1836.152673437.$$

They do not use the non-nucleon $C_{B},\kappa_{B},\lambda_{B},\mu_{B}$ assignments. They are separate read-outs of the same neutral-parent scale, not imported results from another manuscript.

The same formulas give

(170) $${\left(m_{n}-m_{p}\right)}_{\mathrm{rec}}=1.293332153\;\mathrm{MeV},$$

within about 0.36eV of the recommended experimental difference. The comparison is numerically stringent, but Remark 9 is essential: the discrete fibers were recognized with knowledge of the nucleon data, and the calculation is therefore a conditional reconstruction rather than a blind prediction.

### 17.2. Sensitivity to the Inherited Charged-Lepton Scale

The many printed digits in Equation (169) follow deterministically from the inherited structural lepton ratios; they should not be confused with a complete theoretical uncertainty budget. Writing

$$Y_{C}\equiv \frac{m_{C}}{m_{e}}=\frac{1+r_{\mu }+r_{\tau }}{2}F_{C},\;C\in \left\{n,p\right\},$$

with $r_{l}=m_{l}/m_{e}$, and holding the discrete nucleon fibers fixed, gives the transparent sensitivity relation

(171) $$\delta Y_{C}=\frac{F_{C}}{2}\left(\delta r_{\mu }+\delta r_{\tau }\right),\;\frac{\partial Y_{C}}{\partial r_{\mu }}=\frac{\partial Y_{C}}{\partial r_{\tau }}=\frac{F_{C}}{2}.$$

Thus, the nucleon agreement directly tests both the inherited lepton scale and the discrete baryon factors. A future uncertainty analysis must separately quantify uncertainty in the lepton reconstruction, truncation of the closure tower, and the physical correspondence postulates. Until that is done, the final digits and $z_{\exp}$ values in Table 5, Table 7 and Table A2 are best read as reproducibility diagnostics under the declared premises.

### 17.3. Experimental Centroid Convention

The structural operator returns one centroid for each isospin multiplet, so the experimental charge states must first be reduced to the same quantity. The following convention keeps that comparison transparent and propagates the quoted charge-state uncertainties without using them in the theoretical construction.

For a multiplet with measured charge states $M_{i}$, the comparison centroid is

(172) $${\bar{M}}_{\exp}=\frac{1}{n}\sum_{i}M_{i},\;\sigma_{\bar{M}}=\frac{1}{n}\sqrt{\sum_{i}\sigma_{i}^{2}}.$$

The inputs follow the PDG 2024 review and 2025 update [3]. The broad Δ(1232) is represented by 1232±2 MeV as a conservative centroid proxy. Because this resonance convention is not statistically equivalent to the precision uncertainties of stable states, the leading comparison emphasizes absolute and relative residuals rather than a combined significance statistic.

### 17.4. Primary Leading-Order Centroid Comparison

Table 6 evaluates Equation (167) before any higher residue is introduced.

**Remark** **10** (Leading truncation)**.** *All seven non-nucleon centroids have absolute relative residuals below 0.4% before any residue operator is applied. This tests the scale and gross operator spectrum separately. The precision comparison belongs to the level-six conditional result below.*

### 17.5. Reconstructed Centroid Spectrum Through Level Six

Table 7 evaluates the spectrum of ${\hat{M}}_{\mathrm{cent}}^{\left(6\right)}$. The operator calculation is exact once its finite exposure complex is fixed. Because the small ledger historically guided discovery of that complex, the table is a stringent reproducibility and internal-consistency test rather than an overdetermined or blind fit-independent prediction; the corresponding information count appears in Table 3.

All seven centroids lie within one quoted comparison uncertainty. This statement concerns empirical consistency under the declared conditional premises; it does not convert experimental error bars into a theoretical uncertainty estimate.

## 18. Why the Full Intrinsic Mass Tower Can Be Alpha-Free

The construction motivates a stronger conjecture than an alpha-free leading centroid followed by necessarily alpha-dependent structural corrections. Every higher term in Equation (137) is intended to refine the same confined closure. Its ingredients are the common scale $R^{2}$, finite interface capacities, protected quotients, orientation pairing, and integer signed residues. At the displayed level, the leading, centroid-residue, and charge-source coefficients are spectra or boundaries of explicit finite operators. The remaining conjectural step is not the level-six calculation but its extension to the complete infinite refinement tower.

**Conjecture** **1** (Alpha-free saturated baryon spectrum)**.** *For every isolated light-baryon state B,a, the complete intrinsic rest mass is the saturated reconstruction invariant*

(173) $$M_{B,a}^{\mathrm{sat}}=R^{2}\left[1-\frac{1}{357}+\frac{C_{B}}{64}+\sum_{n=4}^{\infty }\frac{C_{\mathrm{res}}^{\left(n\right)}\left(B\right)+C_{\mathrm{orient}}^{\left(n\right)}\left(B,a\right)}{64D_{4}D_{5}\cdots D_{n}}\right],$$

*with the dedicated proton and neutron ground-state formulas replacing the generic N entry. The displayed coefficients through level six are supplied by Equations* (116)*,* (125) *and* (137) *under the declared finite-complex and carrier-lift premises. The conjecture is that the same closure principles select all higher coefficients for n≥7. The fine-structure constant is not introduced as an independent mass input.*

This conjecture does not claim that QED calculations of electromagnetic contributions are incorrect. It distinguishes explanatory levels. Effective field theory can write the completed mass as a sum of QCD, QED, and quark-isospin pieces. The reconstruction theory instead asks whether the total isolated mass is selected as one complete admissible state. The two descriptions can agree numerically while organizing the explanation differently.

A conventional effective-theory derivative $\partial M_{B}/\partial \alpha$ remains meaningful within a specified QCD + QED parameterization. The reconstruction proposal makes a different claim: α and $M_{B}/R^{2}$ may be correlated read-outs of common premetric data, so independent variation of one read-out while holding all reconstruction data fixed need not represent an admissible structural deformation. Establishing a quantitative matching between these descriptions remains an open problem. The operational claim tested here is narrower: the displayed structural mass relations do not use measured α as an input.

### Why the Present Selection Question Is Not Formulated at the Lagrangian Level

The Lagrangian framework begins after the relevant spacetime, field content, representations, and couplings are specified. It can calculate how an identified baryon propagates, interacts, and decomposes into strong, electromagnetic, and quark-isospin contributions in a chosen scheme. The proposed premetric questions—codimension-two persistence, protected identity content, bilateral admissibility, projective saturation, and the finite exposure ledger—are not coupling-expansion questions about an already selected state. They therefore require a separate reconstruction description if the premises of this paper are adopted.

This distinction does not lower the standard of rigor; it changes the form that rigor must take at the earlier level. The appropriate requirements are explicit finite objects, complete invariant quotients, exhaustive admissibility checks, uniqueness or no-go theorems, and read-out maps that can be tested without choosing among alternatives by their numerical agreement. A later matching to QCD is also necessary for a mature theory, but that downstream matching is different from the upstream selection of the finite carrier complex.

The appropriate division of labor is therefore

(174) $$\begin{array}{cc} \; & \mathrm{reconstruction}\;\mathrm{selects}\;\mathrm{the}\;\mathrm{complete}\;\mathrm{state},\\ \; & \mathrm{the}\;\mathrm{Lagrangian}\;\mathrm{describes}\;\mathrm{its}\;\mathrm{effective}\;\mathrm{dynamics}. \end{array}$$

The conjecture is falsified if the finite carrier fibers cannot be obtained from a complete premetric reconstruction calculus, if arbitrary noninteger or α-dependent patches are required, or if new precise masses exhibit a systematic residual that cannot be generated by the same intrinsic orientation complex.

## 19. Relation to Other Work

The proposal touches several established research traditions but should not be identified with any one of them. This section clarifies the overlap and the differences.

### 19.1. Quantum Chromodynamics and Lattice Calculations

QCD remains the tested continuum theory of color dynamics. It describes quark and gluon fields, asymptotic freedom, confinement, scattering, hadronization, and the nonperturbative spectrum. In the standard account, most of the nucleon mass is generated dynamically rather than obtained by adding current-quark masses; the renormalized energy–momentum tensor and its trace anomaly provide the appropriate QCD mass decomposition [18]. Lattice QCD supplies a first-principles numerical route from the gauge theory, quark masses, and scale setting to the light-hadron spectrum [1,2,4,19]. Nothing in the present construction disputes dimensional transmutation, the trace anomaly, or the lattice spectrum.

The proposed claim is instead a matching hypothesis between explanatory levels. A premetric closure calculus is asked to select the admissible primitive channel content and dimensionless placement of the completed light-baryon states; QCD is then the indispensable post-read-out dynamical theory of those states. In particular, the appearance of the charged-lepton-derived scale $R^{2}$ does not mean that lepton masses dynamically cause baryon masses. It asserts a cross-sector structural normalization that must ultimately be matched to the QCD scale. No such explicit cross-level matching is proved here, and failure to obtain one would count against the reconstruction interpretation.

The finite $Z_{3}$ notation must therefore be read carefully:

(175) $$Z_{3}\;\mathrm{closure}\neq SU{\left(3\right)}_{C}\;\mathrm{Yang–Mills}\;\mathrm{theory}.$$

The first is a proposed pre-read-out completeness condition; the second is the established effective gauge theory. Topological ideas also occur inside conventional gauge theory and confinement studies [20], but the present use of codimension-two linking concerns the support of persistent identity rather than a derivation of the QCD mass gap.

### 19.2. QCD + QED Mass Decompositions

Modern QCD + QED lattice calculations can separate strong-isospin and electromagnetic contributions under specified renormalization and matching conventions, including the neutron–proton splitting [21]. The decomposition is physically essential and scheme-aware; it is not challenged by the present proposal. Conversely, agreement of a total-mass structural formula does not reproduce that decomposition, a scattering amplitude, or a response to continuously varied quark masses and couplings.

The narrower statement made here is that measured α is not inserted as an independent numerical input into the displayed reconstruction formulas. It is not a claim that electromagnetic interaction energy vanishes, that the physical masses are independent of QED, or that $\partial M_{B}/\partial \alpha$ is meaningless in a specified effective parameterization. Establishing an explicit matching map from the reconstruction residues to QCD+QED mass components is a major open test.

### 19.3. Flavor Symmetry, the Quark Model, and Baryon Mass Relations

The octet and decuplet organization is standard and historically motivated by approximate flavor SU(3), the Eightfold Way, and Gell-Mann–Okubo-type relations [22,23]. Once a three-dimensional space *W* and symmetry of two tensor roles are given, the identity ${\mathrm{Sym}}^{2}\left(W\right)⊗W≅{\mathrm{Sym}}^{3}\left(W\right)⊕S_{\left(2,1\right)}\left(W\right)$ is precisely the familiar 10+8 representation combinatorics. The contribution of Section 8 is therefore not an independent derivation of the observed multiplet multiplicities. It constructs a rooted bilateral route complex whose quotient linearizes into the same representation pattern and localizes the physical step in the explicit correspondence $W\to 3_{F}$.

The mass-exposure ledgers then use the observed family labels when assigning centroid weights. The proposed interpretation is that the route complex provides an upstream structural realization of known flavor organization; the correspondence postulate and the data-informed exposure fibers still carry the physical burden. No claim is made to derive QCD flavor dynamics, quark charges, or symmetry-breaking effects.

The approximately equal decuplet spacing is represented here by repeated two-sided continuation exposure. This is a structural reinterpretation of a known regularity, not a denial of the usual explanation in terms of flavor-symmetry breaking and QCD dynamics. A successful mature theory would need to show how the closure labels match, constrain, or emerge from the effective flavor description.

### 19.4. Topological Defects, Holonomy, and Reconstruction

The use of complement topology and linking loops is standard in the classification of defects and gauge holonomy [24,25,26]. What is nonstandard is the application of these tools to particle identity and mass read-out. The author’s International Journal of Topology paper developed a classification of admissible reconstruction operations and identified codimension two as the critical support for persistent loop-detectable obstruction [7]. The later carrier-closure paper applied that result to particle ontology, twofold Lorentz read-out, nested three-sector closure, and the projective origin of the leading generation count [6].

The present baryon paper is downstream from both works. It does not repeat the entire topological classification. Instead, it uses the codimension-two link, projective grades, protected identity, and read-out seam to justify the finite denominators entering the baryon centroid calculation.

### 19.5. Emergence and Effective Field Theory

The distinction between structural organization and effective continuum dynamics is compatible in spirit with the broader idea that different descriptive levels can possess autonomous variables and laws [27,28]. Effective field theory likewise separates low-energy observables from unresolved short-distance degrees of freedom and organizes corrections according to scale and symmetry [29]. The present proposal differs by assigning a specific premetric closure architecture beneath the effective fields. Its claims therefore exceed generic emergence and must be tested through the discrete comparisons and theorem targets stated in this paper.

The tower in Section 12 resembles an effective expansion in that deeper refinements are increasingly suppressed. It is not, however, a conventional perturbation series in a coupling or momentum ratio. The suppression factors are interpreted as conditional interface capacities. Establishing a systematic matching between this tower and a standard effective field theory remains future work.

### 19.6. Compositeness and Preon Models

Preon and compositeness models seek smaller material constituents of quarks and leptons [30,31]. The present construction takes the opposite route. A protected chamber, a $Z_{n}$ role, or a latent continuation seam is not a new subparticle with its own ordinary position and binding interaction. These are relational components of one closure complex. The framework therefore avoids multiplying unobserved constituents, but it assumes a nonstandard reconstruction ontology that must earn support through successful derivations.

### 19.7. Structural Mass Relations and Numerology

The use of simple dimensionless relations in particle masses has a long history, including the Koide charged-lepton relation [12]. Numerical simplicity alone is not evidence of a physical mechanism. Here, the displayed family ledgers are no longer free tables of integers: they are the spectra of the finite operators $C,R_{4},R_{5},R_{6}$, and the combined charge ledger is the boundary of the cochains $h_{8},h_{10}$. This makes the construction algebraically reproducible and exposes the precise premises on which it depends.

The published co-selection framework also changes the relevant prior question [9,10]. The same carrier–defect architecture constrains both admissible law forms and finite numerical normalizations, so the present search is not for an arbitrary identity unrelated to the dynamical platform. This continuity makes an isolated numerological coincidence less plausible, but it is not independent validation: the papers share premises, and co-selection does not determine the baryon-specific fibers. The explicit count in Table 3 is therefore still required.

The correspondence risk has not disappeared. The finite carrier fibers were recognized with historical knowledge of the light-baryon spectrum, and saturation alone does not prove that a complete reconstruction calculus must select exactly those fibers. The numerical agreement is therefore a conditional finite reconstruction and an internal-consistency test, not a blind discovery from topology or a statistical prediction. The frozen Delta charge vector in Corollary 2 and any future independent premetric derivation of the same fibers provide genuinely prospective tests.

**Remark** **11** (Position of the Present Proposal)**.** *The framework is complementary to QCD, uses standard topological tools in a nonstandard reconstruction setting, differs from constituent compositeness models, and must be judged by whether the declared finite complexes can be derived independently and then reused without data-driven modification.*

## 20. Falsifiability and Remaining Theorem Targets

The construction is falsifiable at several levels. In this list, “derive” means derive inside a complete premetric incidence and read-out calculus unless an explicit post-read-out QCD comparison is named.

1. **Nucleon-refinement failure.** A complete premetric reconstruction theory must select the binary projective overlap read-out in Structural Postulate 6 independently of the measured nucleon masses. If an equally admissible refinement gives different indices, or if the counts or incidence rule must be retuned as measurements improve, the nucleon construction fails.
2. **Information-weight failure.** If the premetric automorphism group is not transitive on an asserted exposure space, equal weighting cannot be inferred from symmetry alone. Any nonuniform weights must then be independently derived rather than hidden inside a chamber count.
3. **Denominator failure.** The factors 64, 357, 14, and the conditional *n*-tower must follow from the stated reconstruction architecture. If the required scale hierarchy cannot be obtained from the chamber structure, the model fails.
4. **Leading-fiber failure.** If the complete reconstruction calculus does not produce the leading fibers in Structural Postulate 3, or produces many inequivalent saturated fibers with comparable mass spectra, the leading operator has no unique explanatory force.
5. **Exposure-complex failure.** The local signed motifs in Definition 5 and the selectors in Structural Postulate 4 must arise from the declared chamber incidence. A required additional motif, or the failure of the no-extra-term classification in Theorem 8, falsifies the displayed centroid reconstruction.
6. **Carrier-lift failure.** A complete premetric carrier calculus must justify Structural Postulate 5. In particular, a precise Delta charge-resolved spectrum inconsistent with Equation (136) would reject the frozen carrier-source construction.
7. **Alpha-free failure.** If precise out-of-sample baryon masses require explicit, independently inserted α-dependent patches that cannot be represented by the same closure calculus, the saturated-spectrum conjecture is false.
8. **Channel-complex failure.** The eighteen-class orbit theorem fails as a physical model if the complete premetric carrier calculus does not commute with independent grade substitutions as in Equation (49), if an additional protected cross-grade relation exists, or if the two confined sides possess an invariant ordering that prevents the $S_{2}$ quotient.
9. **Flavor-read-out failure.** The structural 10+8 decomposition must admit the light-flavor correspondence Equation (65) without contradicting charge assignments or effective flavor phenomenology.
10. **New-observable failure.** Any later use of the number eighteen must independently prove that the relevant selector factors through $\Sigma_{B}^{\left(0\right)}$ and weights each primitive class once rather than counting spin projections, antiparticles, excitations, or heavy-flavor states.

The immediate theorem targets are:

1. Derive the grade-local commutation relation Equation (49) from a complete premetric carrier calculus and classify any possible protected cross-grade obstruction;
2. Derive the light-flavor representation map Equation (65), including its charge and hypercharge read-outs, rather than imposing it only as a sector correspondence;
3. Prove that higher $Z_{n}$ refinements act internally on $\Sigma_{B}^{\left(0\right)}$ unless they introduce a genuinely new protected identity invariant;
4. Derive the leading, signed-exposure, and carrier-lift fibers directly from the premetric chamber calculus, without historical recognition of the baryon masses;
5. Prove or refute uniqueness of those finite fibers under IRSP saturation and the stated protected relations;
6. Improve the charge-resolved Delta measurements and test the frozen vector Equation (136);
7. Derive the binary-projective overlap read-out in Structural Postulate 6 from the complete premetric incidence complex, prove or refute its uniqueness, and do so without using the measured nucleon masses as a selection criterion;
8. Extend the finite exposure construction to levels n≥7 and test whether the alpha-free tower remains closed;
9. Test the same rules on additional hadronic sectors without introducing new continuous parameters.

## 21. Conclusions

This paper has developed a reconstruction model for both the finite channel content and the structural mass organization of the light-baryon spectrum. The central object is a confined bilateral neutral-parent pocket whose two conjugate sides and protected continuation seam close only as a whole. Codimension two supplies the transverse linking circle required for persistent loop-readable identity, while the longitudinal projective space ${\mathrm{RP}}^{2}$ supplies three leading grades.

The principal finite result is Theorem 3. Within the declared primitive complex, two unordered conjugate-side roles and one distinguished continuation role give the raw route space $G^{3}$, and the complete invariant ledger quotients only the side exchange. Quotient-first saturation therefore yields exactly eighteen route classes. Complex linearization gives

(176) $${\mathrm{Sym}}^{2}\left(W\right)⊗W≅{\mathrm{Sym}}^{3}\left(W\right)⊕S_{\left(2,1\right)}\left(W\right),$$

with dimensions 10+8. Once the three-grade space and side symmetry are posited, this is the familiar flavor-representation combinatorics. The physical identification with the decuplet and octet is imposed by Structural Postulate 2; the theorem therefore supplies a structural realization of known flavor organization, not an independent derivation of the observed multiplets. What remains exact and available for downstream work is the complete primitive kernel $K_{B}=C\left[\Sigma_{B}^{\left(0\right)}\right]$ with dimension 18. Any new selector using that dimension must separately prove that it acts once on each kernel class. The remaining carrier-level obligations are to derive grade-local completion, exclude an additional protected cross-grade constraint, and establish the flavor map independently.

The mass calculation is a distinct read-out of the same pocket. Under the stated orientation-independence assumption, inheritance of all three grades by the two confined sides gives six binary mass-orientation slots and the conditional capacity $2^{6}=64$; the protected continuation seam labels the complete channel identity without becoming a separately exposed scalar-mass constituent. The common scale is the published charged-lepton scale and, by Equation (12), is equivalently the Koide root-amplitude combination in Equation (13); it is not an independent baryon fit.

Premetric equal weighting is not an additional arbitrary numerical rule. When the automorphism group acts transitively on a finite admissible exposure space, invariance forces the uniform distribution. Its maximum Shannon entropy is $H_{\max}=\log N$, so one unresolved chamber carries the inverse information capacity $e^{-H_{\max}}=1/N$. This result explains the inverse-capacity form of the leading deficits; it does not choose the admissible complex, whose derivation remains the harder structural task.

A rooted five-role model gives the conditional ground-state denominator 3(5!−1)=357. The first bilateral refinement gives 2(3+4)=14, and later conditional refinements supply 5,6,7,…. Within this hierarchy, the leading exposure operator C has spectrum (0,12,17,26;20,30,40,50). The signed index operators $R_{4},R_{5},R_{6}$ generate the complete displayed centroid ledger from one finite signed complex rather than from eight unrelated assignments. This algebraic compression does not erase the data-informed selection of the complex. At leading order, two normalization integers and five effective operator weights organize seven centroids; levels 4–6 add nine signed coefficient occurrences for the same seven residuals. The quantitative audit in Table 3 therefore classifies the numerical agreement as retrospective reconstruction rather than predictive surplus.

The neutron and proton are resolved within the same program rather than imported from a separate paper. The closed-neutral and charged-boundary exposure spaces give $N_{n}=470$ and $N_{p}=286$. The binary span of the three projective grades then supplies the unique seven-point Fano plane. Under the overlap read-out in Structural Postulate 6, Theorem 13 calculates

$$\frac{1}{30},\;\frac{3}{7},\;\frac{23}{42}$$

as, respectively, a diagonal neutral overlap, a closed-line screening density, and a line-stabilizer restoration index over oriented flags. These yield $m_{n}/m_{e}=1838.683661321$, $m_{p}/m_{e}=1836.152673437$, and ${\left(m_{n}-m_{p}\right)}_{\mathrm{rec}}=1.293332153\;\mathrm{MeV}$. The discrete fibers were recognized with knowledge of the nucleon data; accordingly, this is a conditional reconstruction and a cross-sector consistency test, not a parameter-free blind prediction. The remaining target is to derive the binary overlap read-out itself from the complete premetric carrier calculus, not to supply a conventional Lagrangian before spacetime and fields have been read out.

The charge-resolved calculation is likewise an operator statement with an explicit retrospective step. Isospin centering proves centroid preservation, while the saturated carrier-source cochains in Theorem 12 have boundaries equal to the unique combined normal form Equation (125). Five coefficients are first determined from six observed non-nucleon edge-flow components and are then realized by the finite carrier fibers. Freezing that construction reproduces the displayed octet and decuplet charge residues and yields the prospective conditional Delta vector (−15,−55,−20,90). The corresponding resolved indices and masses are displayed explicitly in Table A1, Table A2 and Table A3.

The alpha-free saturated-spectrum conjecture is that the complete isolated baryon mass, including deeper and charge-orientation refinements, can ultimately be selected without measured α as an independent input. The published theory and fine-structure papers place this conjecture in a law–constant co-selection program [9,10]. That shared architecture makes the present relations less isolated, but it does not prove the baryon-specific fibers and is not counted as an independent statistical confirmation. Nor does the proposal replace QCD or QED. Those theories remain indispensable for post-read-out propagation, interactions, and mass decomposition.

Numerically, the leading truncation places all seven non-nucleon centroids within 0.4%. The reconstructed level-six centroid operator places all seven within one quoted comparison uncertainty; their experimental-normalized residuals range from −0.21σ to +0.93σ. For the ten resolved non-Delta charge states, nine lie within 1σ and all ten within 1.1σ, using experimental uncertainties only. These comparisons use no continuously adjusted baryon-sector scale and no measured α. Because no theoretical uncertainty has yet been assigned to the correspondence premises or closure-tower truncation, the reported $z_{\exp}$ values are reproducibility diagnostics rather than statistical significances; Equation (171) separately displays the dependence on the inherited lepton ratios.

The result is therefore best described as a conditional structural organization of known light-baryon information together with a retrospective finite reconstruction of the displayed masses. The orbit, representation, exposure-index, and boundary calculations are exact inside their declared complexes; the physical identification and selection of those complexes, especially the mass-exposure and carrier-lift fibers, remain correspondence premises discovered with historical knowledge of the spectrum. This limitation is explicit and testable. QCD and QED remain indispensable for post-read-out dynamics and conventional mass decompositions. A later metric-capacity use of the number eighteen would still require a separate proof that its selector factors through the primitive channel space and weights each class once.

## Figures and Tables

**Table 1:** Compact translation of the reconstruction terminology used in the baryon calculation.

Term	Operational Meaning
**carrier; premetric structure**	non-material support that makes a completed structure readable; its upstream data are incidence, closure, protection, and admissibility rather than metric distance or field energy
**reconstruction; read-out**	recurrent enforcement of an admissible identity; the map representing the completed identity by effective spacetime and particle variables
**defect; closure; neutral parent $P_{0}$**	a protected obstruction readable through its complement; consistent return of the whole structure; the balanced pre-branch closure archetype
**protected content; carrier-facing exposure**	data required for identity but not independently visible; the signed residue that survives quotienting, protection, and orientation pairing
**IRSP**	existence condition requiring identity and observables to descend consistently under every admissible continuation; it does not assert that a particular physical realization is immortal. Indistinguishable representatives are quotiented before channels are counted
**saturation**	inclusion of every inequivalent admissible channel required by closure after the observable quotient; it is neither minimization nor a stopping rule
**$Z_{n}$ interface; bilateral pocket**	an *n*-role finite resolution of one return structure, not a gauge group or polygon; two individually incomplete oriented sides that close jointly, denoted by $\hat{⊕}$
**complete carrier calculus**	a future upstream theory that must select the finite complexes and read-out maps without choosing among them by mass agreement
**alpha-free saturated mass**	a proposed complete structural rest mass with no independently inserted measured α; post-read-out QCD + QED decompositions remain valid

**Table 2:** Previous cross-sector applications of the neutral-parent reconstruction framework. Numerical values, where shown, are those reported in the cited works.

Application	Structural Result and Reported Agreement
Native-law selection [9]	IRSP and the equipped carrier–defect pair conditionally select the Lorentzian read-out platform and the leading Maxwell–Einstein law classes. The result supplies the law side of co-selection, not a baryon mass formula.
Fine-structure capacity [10]	A neutral codimension-two holonomy-capacity construction gives the fine-structure normalization as a distinct read-out of the common architecture. Its value is not inserted into the present baryon formulas.
Nucleon magnetic moments [6]	The closure calculation gives $\mu_{n}/\mu_{p}=-0.684979364944$, differing from the reference experimental ratio by 0.022ppm, or approximately 0.093σ.
Charged-lepton hierarchy [11]	The saturated charged-lepton construction gives $\frac{m_{\mu }}{m_{e}}=206.768282689,\;\frac{m_{\tau }}{m_{e}}=3477.441636,\;\frac{m_{\tau }}{m_{\mu }}=16.8180612.$ The reported deviations are −0.004σ, approximately 0.43σ, and approximately 0.43σ, respectively.

**Table 3:** Quantitative information audit. “Effective discrete ingredients” counts the numerical structures at the displayed operator level, including data-informed choices. The imported lepton scale is a prior cross-sector result and $m_{e}$ is only the reporting unit.

Stage	Compared Information	Effective Discrete Ingredients	Warranted Status
**Primitive channels**	known 10- and 8-dimensional flavor sectors	five structural selections: one three-grade set; one three-role assignment; one $S_{2}$ side quotient; one grade-local completion rule; and one physical flavor correspondence	exact orbit and representation theorem inside the declared complex; structural reorganization of known flavor combinatorics, not an independent prediction of the multiplets
**Leading centroids**	seven non-nucleon centroids	two common normalization integers, 357 and 64, plus five effective leading weights 12,5,2,20,10: seven numerical ingredients for seven values	exactly determined once the data-informed leading fibers are fixed, but not overdetermined; a structural compression and cross-sector consistency test
**Levels 4–6**	the same seven centroids	nine signed coefficient occurrences (−1,5,−3,−7;6,−6,−3;1,−2), containing six distinct magnitudes, together with their motif and projector placement	retrospective finite reconstruction of the residuals; no independent predictive surplus is claimed at this order
**Resolved non-nucleon charges**	six independent zero-sum edge-flow components	five normal-form coefficients 35,161,55,15,25, determined from those flows before their carrier-fiber reinterpretation	one internal cross-multiplet constraint plus a frozen prospective Delta vector; the five fitted coefficients are not presented as blind predictions
**Nucleon closures**	two mass ratios	five numerical ingredients—two exposure counts 470, 286 and three correction fractions—generated by one binary-projective overlap postulate	data-informed conditional reconstruction testing the independently inherited lepton scale; not a parameter-free prediction

**Table 4:** Leading exposure spectrum generated by Equation (100). Its physical status is conditional on Structural Postulate 3.

Family	Exposure Ledger	$C_{B}$	Interpretation
*N*	0	0	Ground asymmetric bilateral pocket
Λ	3(5−1)	12	One protected-singlet strange continuation
Σ	3(5−1)+5	17	Strange core plus isotriplet boundary exposure
Ξ	2·3(5−1)+2	26	Two strange continuations plus bilateral endcap
Δ	$2(3^{2}+1)$	20	Spin-aligned bilateral source–return base
$\Sigma^{*}$	20+10	30	One strange decuplet endpoint
$\Xi^{*}$	20+2·10	40	Two strange decuplet endpoints
Ω	20+3·10	50	Three strange decuplet endpoints

**Table 5:** Conditional nucleon mass-ratio calculation. The comparison values are CODATA recommended values [17]. Here, $z_{\exp}=\left(x_{\mathrm{rec}}-x_{\exp}\right)/\sigma_{\exp}$ uses only the quoted experimental uncertainty and is not a theory significance.

Quantity	Reconstruction	Comparison	Residual	$z_{\exp}$
$m_{n}/m_{e}$	1838.683661321	1838.68366200(74)	$-6.79\times 10^{-7}$	−0.92σ
$m_{p}/m_{e}$	1836.152673437	1836.152673426(32)	$+1.10\times 10^{-8}$	+0.35σ

**Table 6:** Leading-order light-baryon centroid comparison. The formula uses only $M_{0}$, $Q_{B}$, and the spectrum of the conditional leading operator C; no $R_{4},R_{5},R_{6}$ term is included.

*B*	${\bar{M}}_{\exp}$ [MeV]	$C_{B}$	$M_{B}^{\left(0\right)}$ [MeV]	Residual [MeV]	Relative Residual
Λ	1115.683000	12	1115.475977	−0.207023	−0.0186%
Σ	1193.153667	17	1189.036071	−4.117596	−0.3451%
Ξ	1318.285000	26	1321.444240	+3.159240	+0.2396%
Δ	1232.000000	20	1233.172127	+1.172127	+0.0951%
$\Sigma^{*}$	1384.576667	30	1380.292315	−4.284352	−0.3094%
$\Xi^{*}$	1533.400000	40	1527.412503	−5.987497	−0.3905%
Ω	1672.450000	50	1674.532690	+2.082690	+0.1245%

**Table 7:** Conditional centroid reconstruction through level six. The quantity $z_{\exp}=\left(M_{\mathrm{rec}}-M_{\exp}\right)/\sigma_{\exp}$ uses only the quoted experimental uncertainty; no theoretical uncertainty is included.

*B*	${\bar{M}}_{\exp}$ [MeV]	$C_{B}$	$\kappa_{B}$	$\lambda_{B}$	$\mu_{B}$	$M_{B,\mathrm{cent}}^{\left(6\right)}$ [MeV]	Residual [MeV]	$z_{\exp}$
Λ	1115.683±0.006	12	0	1	0	1115.686149	+0.003149	+0.52σ
Σ	1193.153667±0.026493	17	4	0	−2	1193.169448	+0.015781	+0.60σ
Ξ	1318.285000±0.105948	26	−3	0	0	1318.291664	+0.006664	+0.06σ
Δ	1232±2	20	−1	0	0	1232.121269	+0.121269	+0.06σ
$\Sigma^{*}$	1384.576667±0.389530	30	4	0	0	1384.495749	−0.080918	−0.21σ
$\Xi^{*}$	1533.400000±0.340000	40	6	0	0	1533.717653	+0.317653	+0.93σ
Ω	1672.45±0.29	50	−2	0	0	1672.430973	−0.019027	−0.07σ

## Data Availability

All numerical data analyzed are publicly available from the CODATA and PDG sources cited in the manuscript.

## Appendix A. Combined Charge-Orientation Indices

The carrier-source theorem fixes the combined level-six index $K_{B,a}^{\left(6\right)}$, which is the observable combination $30\kappa_{B,a}+6\lambda_{B,a}+\mu_{B,a}$. A decomposition of this combined index into three separate historical columns is neither required nor unique. The mass formula is

$$M_{B,a}^{\left(6\right)}=M_{B,\mathrm{cent}}^{\left(6\right)}+K_{B,a}^{\left(6\right)}Q_{B}^{\left(6\right)}.$$

The complete derived index ledger is as follows:

**Table A1:** Combined charge-orientation indices obtained as boundaries of the carrier-source cochains. Every multiplet sums to zero, so the centroid is preserved exactly.

State	$K^{\left(6\right)}$	State	$K^{\left(6\right)}$	State	$K^{\left(6\right)}$
$\Sigma^{+}$	−108	$\Xi^{0}$	−98	$\Delta^{++}$	−15
$\Sigma^{0}$	−15	$\Xi^{-}$	98	$\Delta^{+}$	−55
$\Sigma^{-}$	123	$\Sigma^{*+}$	−50	$\Delta^{0}$	−20
$\Xi^{*0}$	−45	$\Sigma^{*0}$	−25	$\Delta^{-}$	90
$\Xi^{*-}$	45	$\Sigma^{*-}$	75		

The non-Delta entries are reproduced by Equation (125); the Delta column is the conditional out-of-table consequence of applying the same frozen decuplet operator at $N_{s}=0$.

## Appendix B. Charge-Resolved Conditional Test

Substituting the derived indices in Table A1 into Equation (137) gives the charge-resolved comparison in Table A2. The quantity $z_{\exp}$ uses the quoted experimental uncertainty only as a scale for the residual; no theoretical uncertainty is included. The finite boundary calculation is exact under Structural Postulate 5, but the carrier fibers were historically identified with knowledge of the non-Delta spectrum. The table is therefore an internal conditional test rather than a blind prediction.

**Table A2:** Charge-resolved level-six conditional calculation. The uncertainties and $z_{\exp}$ values are experimental only.

State	Reconstruction [MeV]	Experiment [MeV]	Residual [MeV]	$z_{\exp}$
$\Sigma^{+}$	1189.386357	1189.37±0.07	+0.016357	+0.23σ
$\Sigma^{0}$	1192.644018	1192.642±0.024	+0.002018	+0.08σ
$\Sigma^{-}$	1197.477968	1197.449±0.030	+0.028968	+0.97σ
$\Xi^{0}$	1314.858860	1314.86±0.20	−0.001140	−0.01σ
$\Xi^{-}$	1321.724469	1321.71±0.07	+0.014469	+0.21σ
$\Sigma^{*+}$	1382.744318	1382.83±0.34	−0.085682	−0.25σ
$\Sigma^{*0}$	1383.620033	1383.7±1.0	−0.079967	−0.08σ
$\Sigma^{*-}$	1387.122895	1387.2±0.5	−0.077105	−0.15σ
$\Xi^{*0}$	1532.141366	1531.80±0.32	+0.341366	+1.07σ
$\Xi^{*-}$	1535.293941	1535.0±0.6	+0.293941	+0.49σ

Nine of the ten resolved non-Delta states lie within one quoted comparison uncertainty and all ten lie within 1.1 times their quoted uncertainties. These experimental-normalized residuals quantify the agreement of the frozen conditional construction; they do not measure uncertainty in the correspondence postulate.

The same operator gives the following Delta charge-resolved values without introducing a new coefficient:

**Table A3:** Conditional Delta charge-resolved predictions obtained from Equation (136) and $M_{\Delta ,\mathrm{cent}}^{\left(6\right)}=1232.121269$ MeV. No $z_{\exp}$ values are assigned because sufficiently precise charge-specific Delta pole masses are not used here.

State	$M^{\left(6\right)}$ [MeV]
$\Delta^{++}$	1231.595840
$\Delta^{+}$	1230.194695
$\Delta^{0}$	1231.420696
$\Delta^{-}$	1235.273844

These four numbers are frozen prospective consequences of the declared carrier-lift complex. Improved charge-resolved Delta pole determinations can therefore test the construction without altering its integer fibers.
